# Broadband, Polarization-Sensitive, and Self-Powered High-Performance Photodetection of Hetero-Integrated MoS_2_ on Lithium Niobate

**DOI:** 10.34133/research.0199

**Published:** 2023-07-20

**Authors:** Zhigang He, Heyuan Guan, Xijie Liang, Junteng Chen, Manyan Xie, Kaiwen Luo, Ran An, Liang Ma, Fengkai Ma, Tiefeng Yang, Huihui Lu

**Affiliations:** ^1^ Guangdong Provincial Key Laboratory of Optical Fiber Sensing and Communications, Jinan University, Guangzhou 510632, China.; ^2^ Key Laboratory of Optoelectronic Information and Sensing Technologies of Guangdong Higher Education Institutes, Jinan University, Guangzhou 510632, China.; ^3^ Institute of Fluid Physics, China Academy of Engineering Physics, Mianyang 621900, China.; ^4^ College of Physics and Electronic Engineering, Hengyang Normal University, Hengyang 421008, China.

## Abstract

High-performance photodetectors hold promising potential in optical communication and imaging systems. However, conventional counterparts are suffering narrow detection range, high power consumption, and poor polarization sensitivity. Characteristics originating from switchable polarization in ferroelectrics can be used to optimize the photo-to-electric procedure and improve the photodetection performance. In this regard, we constructed a configuration by integrating 2-dimensional molybdenum disulfide (MoS_2_) with ferroelectric lithium niobate (LiNbO_3_), resulting in the MoS_2_/LiNbO_3_ heterostructured photodetector. Benefiting from the pyroelectric effect of LiNbO_3_, the limitation of bandgap on the detection range can be broken, thus broadening the response band of the detector to 365 to 1,064 nm, as well as enabling the self-powered characteristic. Meanwhile, high carrier mobility and decent light absorbance of MoS_2_ introduce robust light-matter interactions with the underlying LiNbO_3_, leading to ultrafast rise/fall times of ≈150 μs/250 μs and switching ratios of up to ≈190. Moreover, the highest responsivity, specific detectivity, and external quantum efficiency achieved were 17.3 A·W^−1^, 4.3 × 10^11^ Jones, and 4,645.78%, respectively. Furthermore, because of the anisotropy of the spontaneous-polarized LiNbO_3_ substrate, the photocurrent of the device achieved a dichroic ratio of 7.42, comparing favorably to most MoS_2_-based photodetectors. This work demonstrates the integration potential between ferroelectric LiNbO_3_ and 2-dimensional materials for high-performance photodetection.

## Introduction

High-performance photodetectors offer tremendous potential in various applications, such as optical communication [1–4], optical imaging systems [5–7], medical diagnostics [8,9], and environmental monitoring [10,11]. Recently, 2-dimensional (2D) materials, particularly layered transition metal dichalcogenides, have attracted widespread attention in the field of integrated photodetectors due to their excellent photoelectric properties [12–15]. Among these materials, MoS_2_ stands out for its weak dark current, high stability, adjustable bandgap of 1.2 to 1.8 eV, high carrier mobility of 200 cm^2^·(V·s)^−1^, and strong light absorbance in the spectral range of 385 to 670 nm [16–18], representing the most reliable 2D layered semiconductor with excellent comprehensive optoelectronic properties [2], making it a frequently preferred option for utilization in optoelectronic devices such as photodetectors, field-effect transistors, and synaptic transistors [19,20]. Nevertheless, in conventional MoS_2_-based detectors, additional operating voltages are required to drive the directional movement of free carriers to form the photocurrent [21–25], while the detection range is restricted by the bandgap value [26,27], and such detector presents negligible response to the polarized illumination owing to the intrinsic isotropy of the MoS_2_ lattice [28,29], thereby impeding the MoS_2_ photodetectors toward low power consumption, broadband, and polarization sensitive. To improve the performance, Lv et al. [30] exploited the ferroelectric nature of poly(vinylidene fluoride-trifluoroethylene) in intimate contact with MoS_2_, engineering the carrier doping in MoS_2_ through their reversible polarization by an external poling field, reaching 11.9 A·W^−1^ at 365 to 532 nm. Except for poly(vinylidene fluoride-trifluoroethylene), LiNbO_3_ is another important ferroelectric crystal possessing remarkable electro-optical characteristics such as high pyroelectric coefficient of −4 × 10^−5^ C·(K·m^2^)^−1^, a wide light transmission range of 350 to 5,000 nm, and a strong photorefractive effect [31–34]. Currently, LiNbO_3_ has been widely used in integrated optoelectronic devices, such as active photonics, modulators, and optical frequency comb [35–37]. However, LiNbO_3_-based photodetectors have received less attention because of the inherent drawback of insulating behavior, such as low conductivity and huge bandgap, which restricts the construction of LiNbO_3_-based photodetectors and hinders the development of optoelectronic integration based on the LiNbO_3_ platforms.

To address these issues, Sun et al. [38] used a high-energy ion implantation setup to plant both plasmonic silver (Ag) nanoparticles and Ag ions into the surface of lithium niobate on insulator thin film and observed the localized surface plasmon resonance effect to benefit the photodetection behavior of the insulating LiNbO_3_, achieving a responsivity of 0.25 A·W^−1^ and a response time of 16 ms. Besides, assembling with 2D photosensitive materials through van der Waals (vdW) integration is another novel approach to exploiting the application of LiNbO_3_ in the field of photodetection [39–41]. In our previous work, the pyroelectric characteristic of x-cut LiNbO_3_ was used to achieve simultaneous n- and p-doping in graphene, forming a homojunction in the zero-gap graphene channel, thus leading to a high sensitivity (≈2.92 × 10^6^ A·W^−1^) and fast response (rise/fall time of ≈23 ms/23 ms) photodetector [39]. Whereas the weak light absorbance of graphene (≈2.3% for single-layer graphene) and large dark current greatly limited the graphene-based detector toward high switching ratio [21,42–44], the polarization detection of incident light is not integrated into the lithium-niobate-based photodetector.

In this work, we conceived and constructed a MoS_2_/LiNbO_3_ heterojunction photodetector to effectively overcome the challenges mentioned above. The ferroelectric polarization of x-cut LiNbO_3_ can be tuned upon external laser illumination, namely, the pyroelectric effect, thus leading to the asymmetrical doping in the MoS_2_, redefining the carrier distribution in the channel, and the generated internal electric field enables self-powered operation without additional bias voltage. Drawing upon the pyroelectric effect of LiNbO_3_ over extraordinarily wide spectral extent [45–48], the limitation imposed by semiconductor bandgap on detection range can be broken through to 365 to 1,064 nm. Concurrently, the inherent shortcomings of pristine LiNbO_3_ can be compensated by integration with MoS_2_, the high mobility and high light absorption characteristics of the latter unit help to improve the performance of the whole heterojunction device to a minimum rise/fall time of ≈150 μs/250 μs, a maximum switching ratio of ≈190, as well as the exceptional obtainable responsivity, specific detectivity, and external quantum efficiency (EQE) of 17.3 A·W^−1^, 4.3 × 10^11^ Jones, and 4645.78%, respectively. In addition, ferroelectric LiNbO_3_ is sensitive to polarized light because of its anisotropic crystal arrangement [49–53], and this polarization detection ability can be inherited to heterojunction detectors through pyroelectric effect, resulting in a dichroic ratio up to 7.42 in self-powered mode, surpassing most previously reported configurations that integrated MoS_2_ with anisotropic GeSe [54], ReSe_2_ [55], and TiS_3_ [56]. This work exhibits a distinctive complementary advantage of integrating ferroelectric LiNbO_3_ and 2D layered semiconductor materials through vdW integration to realize high-performance photodetection, which can pave the way for the on-chip optoelectronic integration based on the LiNbO_3_ platform.

## Results and Discussion

### Device fabrication

**Fig. 1.**
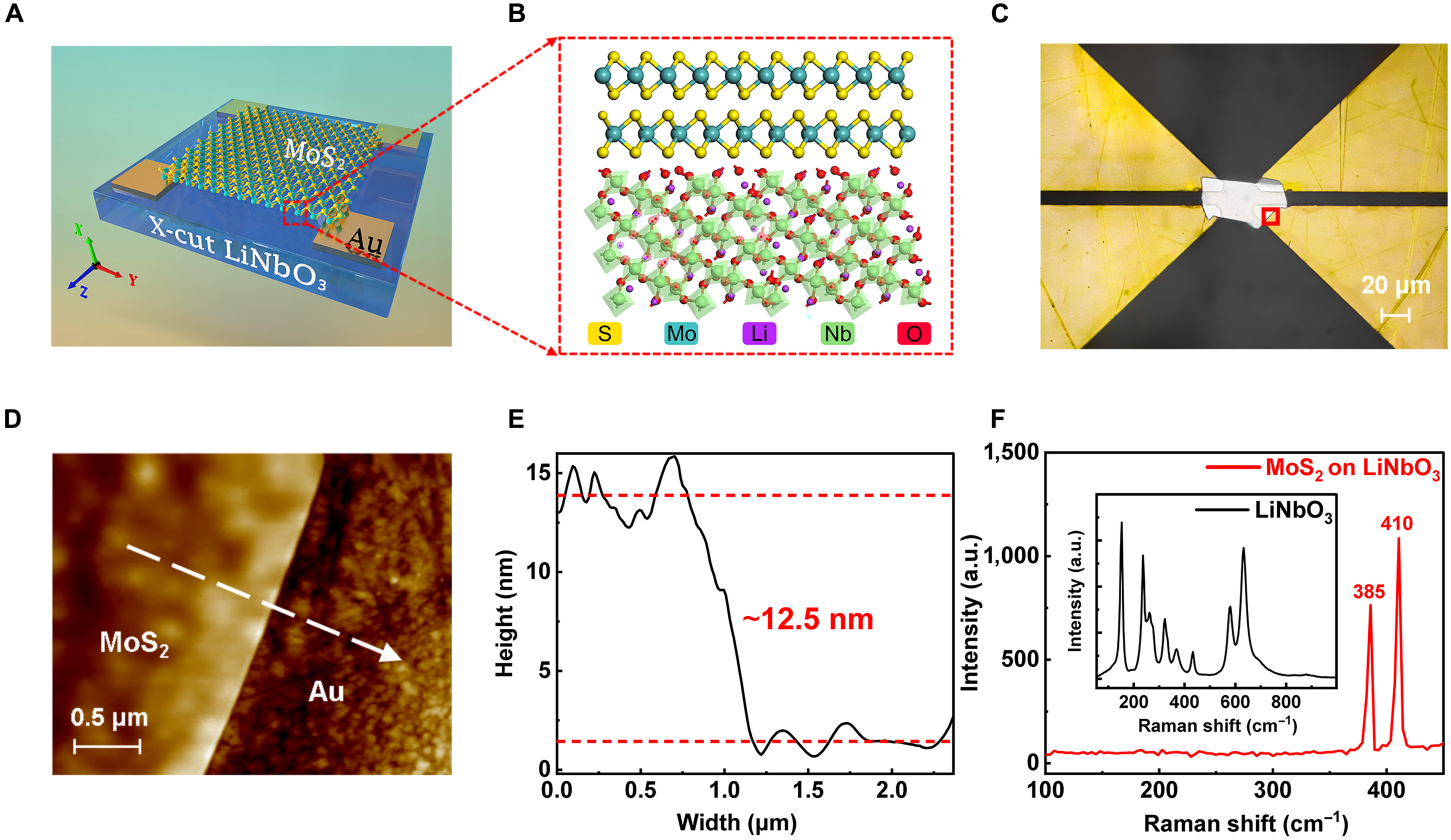
Device configuration and characterizations. (A) Schematic of the MoS_2_/LiNbO_3_ photodetector. (B) Cross-sectional illustration of the atomic structure. (C) Optical microscope image of the fabricated device. (D) AFM topography image of the device collected from the red square area in (C). (E) Height profile of the MoS_2_ obtained by AFM. (F) Raman spectra of the MoS_2_ on LiNbO_3_. Inset is the Raman spectra of the x-cut LiNbO_3_ collected from the same device. a.u., arbitrary units.

Figure 1A shows the schematic structure of a MoS_2_/LiNbO_3_ device, in which a piece of x-cut LiNbO_3_ is used as the substrate, while 2D MoS_2_ works as the channel material. In our case, 2 pairs of electrodes are designed and patterned parallel to the *z*-axis direction of the LiNbO_3_ substrate, since the structural anisotropy of LiNbO_3_ is largest along the *z* axis [32], which is beneficial to maximize the ferroelectric polarization characteristics of LiNbO_3_. To utilize the high conductivity of MoS_2_ to compensate for the poor conductivity of LiNbO_3,_ we selected mechanically exfoliated multilayer MoS_2_ due to its low defect concentration. More details about the fabrication process can be seen in Materials and Methods and Fig. S1. Figure 1B depicts the cross-sectional atomic structure of the MoS_2_/LiNbO_3_ heterojunction, from which we can see that the surface of 2D MoS_2_ is atomically flat and there are no unsaturated dangling bonds, which ensures its compatibility with LiNbO_3_ and can be used to construct devices through vdW integration. As for the underlying LiNbO_3_ crystal, niobium (Nb) and lithium (Li) will move in the same direction along the *c* axis, Nb deviates from the center of the oxygen octahedron, while Li deviates from the common plane of the oxygen octahedron, and an electric dipole moment along the *c* axis is generated, resulting in the spontaneous polarization characteristic [57]. Figure 1C presents the optical microscope image of the real MoS_2_/LiNbO_3_ device, where a mechanically exfoliated MoS_2_ with lateral size of ≈30 μm × 40 μm is evenly laid between 4 Au electrodes and in good contact with them, and the separation between the upper and lower electrodes is ≈10 μm, while the left and right electrodes are spaced ≈20 μm apart. To investigate the roughness of the achieved MoS_2_/LiNbO_3_ device, we characterized and tested the MoS_2_ using an atomic force microscope (AFM), as shown in Fig. 1D. The AFM image is acquired from the red square area in Fig. 1C of the device, it can be observed that the boundary between MoS_2_ and the Au electrode is clear. Figure 1E displays the height line profile across the boundary, indicating that the thickness of MoS_2_ obtained is ≈12.5 nm and the noticeable difference in thickness between the MoS_2_ and the underlying Au electrode indicates a close and uniform contact (more details about the microstructure can be seen in Figs. S2 and S3). Besides, the Raman spectrum was used to characterize the quality of the MoS_2_ on x-cut LiNbO_3_ (Fig. 1F). Under the excitation of a 532-nm laser, 2 prominent peaks around 385 and 410 cm^−1^ are observed, which correspond to the in-plane E1 2g mode and the out-of-plane A_1g_ mode of MoS_2_, respectively [58]. The lowest frequencies at 152 and 238 cm^−1^ in the Raman spectra of the x-cut LiNbO_3_ are mainly due to the deformation of the Nb–O framework. Nb motions appear below 300 cm^4^ Frequencies within the range of 270 to 400 cm^−1^ are affected by the displacement of Li cation, whereas the bending modes of O–Nb–O are observed below and at 432 cm^−1^ and are strongly correlated with the Li–O stretching and O–Li–O bending modes. The range of 550 to 670 cm^−1^ is associated with Nb–O stretching modes involving shifts of oxygen atoms [59,60]. More importantly, all the characteristic peaks show no substantial shift, indicating that the material characterization remains unchanged after the preparation of the MoS_2_/LiNbO_3_ heterojunction, which demonstrates the high quality of the heterojunction.

### Photoresponse of MoS_2_/LiNbO_3_ photodetector

**Fig. 2.**
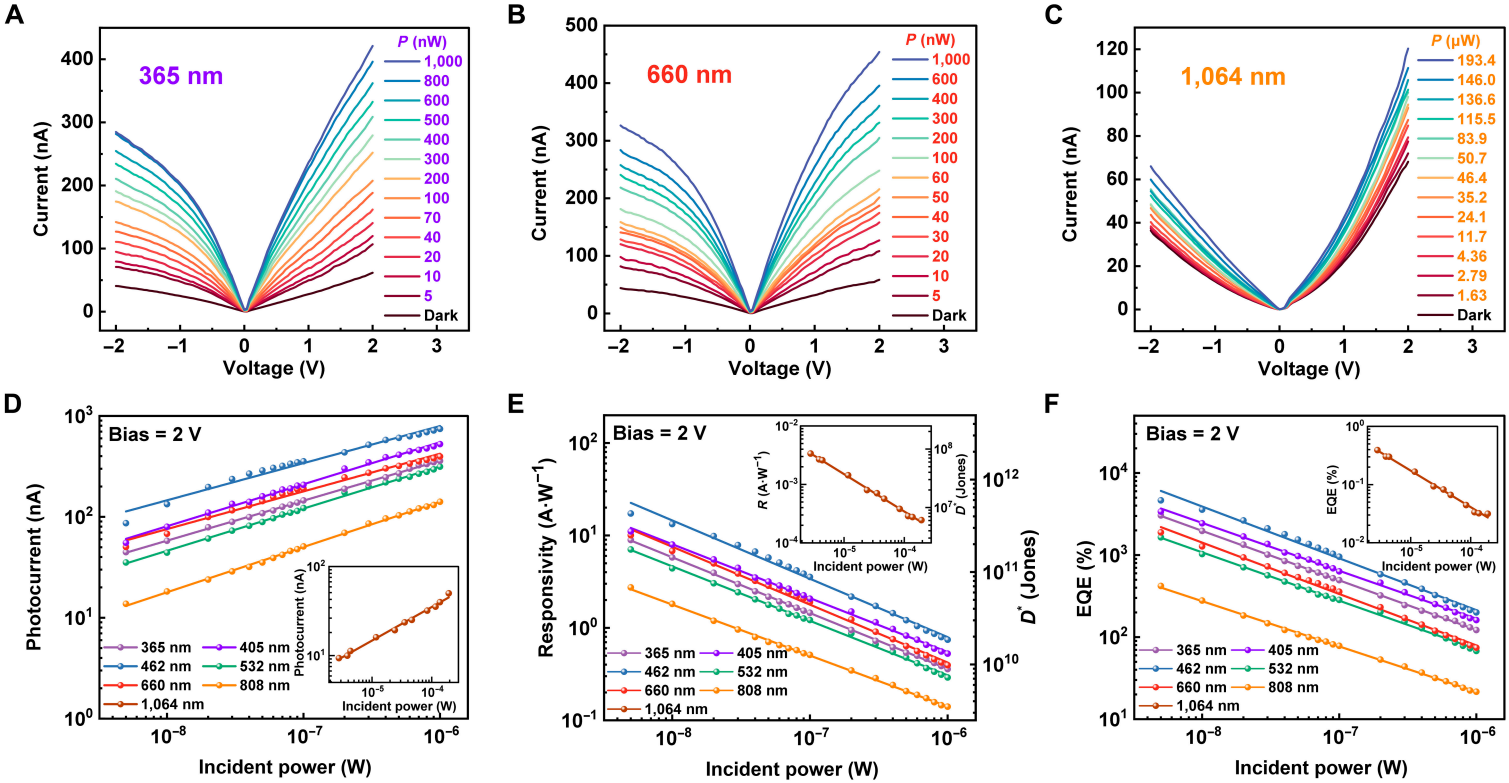
Characteristics of the MoS_2_/LiNbO_3_ photodetector. *IV* curves of the MoS_2_/LiNbO_3_ photodetector illuminated by (A) 365-nm, (B) 660-nm, and (C) 1,064-nm lasers, respectively. (D) Extracted photocurrent as a function of incident power (ultraviolet to NIR). (E) Comparison of D* and *R* under different laser irradiation. (F) Comparison of EQE under different laser irradiation.

The photoresponse behavior of the achieved MoS_2_/LiNbO_3_ device is firstly examined. As shown in Fig. 2A, upon irradiation of a 365-nm laser, the current dramatically increased and monotonically rose to more than 421.1 nA at 1-μW incident power, exhibiting an apparent photosensitive characteristic. As for the situation of 660-nm laser irradiation, the current gradually increases from 58.5 to 454.2 nA, as the power increases from 5 nW to 1 μW (Fig. 2B). In addition to showing good photoresponse in the ultraviolet and visible bands (see Fig. S4), the device even exhibits marked photoelectric response under light irradiation with energy less than the bandgap of MoS_2_, extending the response range to near-infrared (NIR) band (1,064 nm), which is absent in pristine MoS_2_ device, as shown in Fig. 2C. The photocurrent (*I*_ph_) that can be expressed by *I*_ph_ = *I*_light_ − *I*_dark_ is a very intuitive physical quantity used to describe photoelectric detection capability, and the photocurrent as a function of the incident power is plotted in Fig. 2D, showing very good linear characteristics in the double exponential coordinate system under laser irradiation covering ultraviolet-visible-NIR, and the relationship between *I*_ph_ and *P*_in_ can be fitted with power exponentials (*I*_ph_ ∝ *P*_in_^α^) within over 97% coefficient of determination, where the maximum α ≈ 0.45 (see Table S1). Moreover, the extracted photocurrent can be further used to calculate the photoresponsivity (*R*) and specific photodetectivity (D*), which follow the equations of *R* = *I*_ph_ / *P*_in_ and D* = *R*$\sqrt{A/2eI_{\text{dark}}}$, respectively. Here, *A* is the effective illumination area of the conductive channel, *e* is the electron charge, and *λ* is the irradiation wavelength (see Fig. 2E). Furthermore, EQE = *Rhc* / *eλ* was also calculated, as shown in Fig. 2F, and it can be seen that *R*, D*, and EQE decrease exponentially with the increase in light intensity due to larger recombination and scattering probability under strong light illumination than that under weak light illumination [61], which is consistent with the previous reports on MoS_2_-based photodetectors. Specifically, at a bias voltage of 2 V, a wavelength of 462 nm, and a low incident power of 5 nW, the device achieves an *R* of 17.3 A·W^−1^, a D* of 4.3 × 10^11^ Jones, and an EQE of 4,645.78%, demonstrating the device’s terrific sensitivity at weak incident power. The evolution of photocurrent as a function of incident laser wavelength can be seen in Fig. S5, and 2 distinct peaks located at 462 and 660 nm can be attributed to the C– and A– exciton related absorption of MoS_2_, respectively [62,63]. The linear dynamic range (LDR) is another critical parameter used to evaluate the performance of a detector, which can be expressed by LDR = 20 log(*P*_sat_ / *P*_low_), where *P*_sat_ and *P*_low_ correspond to the highest and lowest incident intensities at which the photocurrent–power curve deviates from the linear range. Under 808-nm illumination, the device achieves an LDR of ≈84 dB (as shown in Fig. S6), further emphasizing its high performance.

### Response time of MoS_2_/LiNbO_3_ photodetector

**Fig. 3.**
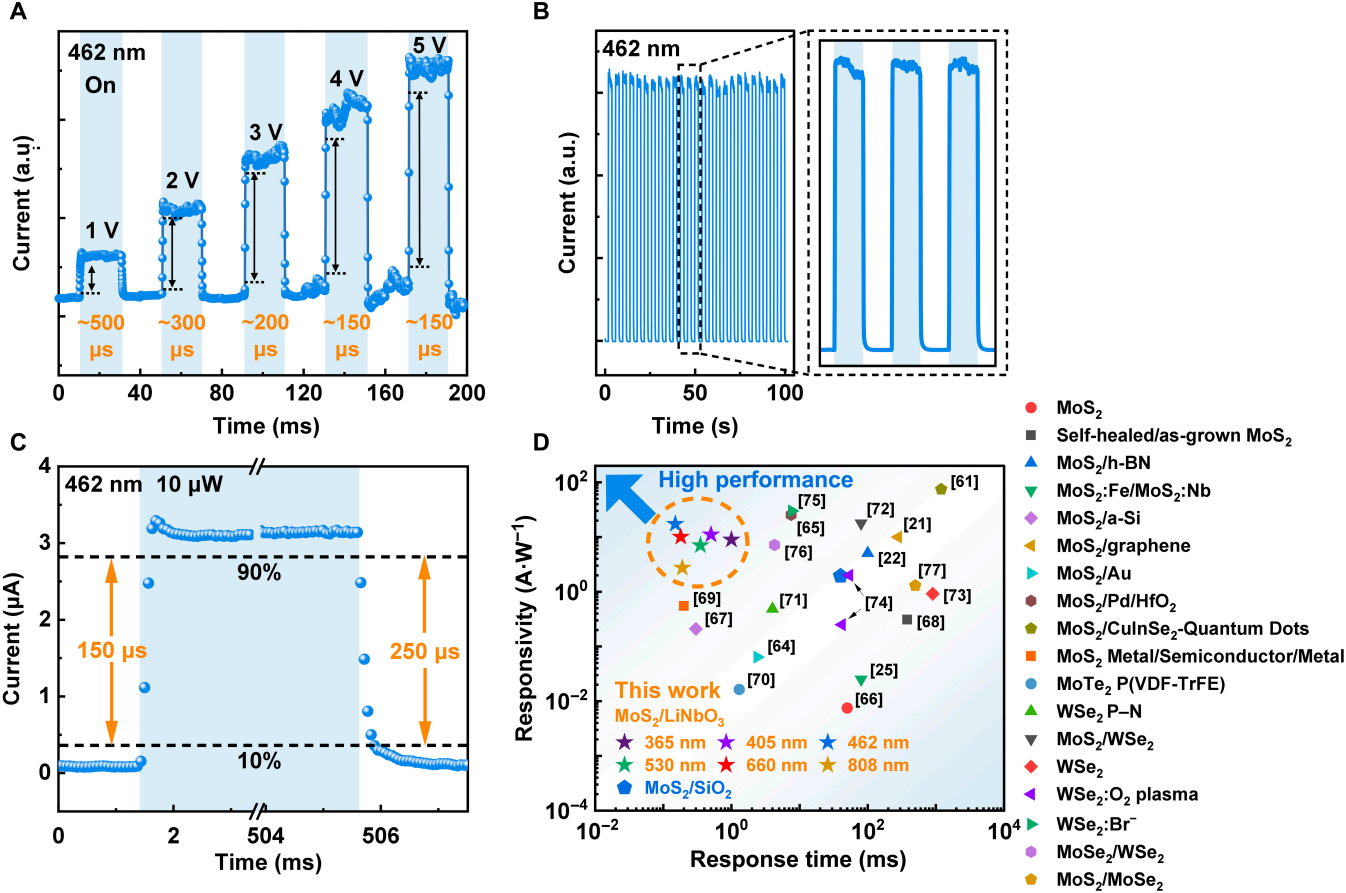
Characterizations of the light-induced switching behavior. (A) Response time of the device at different biases. (B) Device stability under periodic irradiation of 462-nm laser (25 cycles). The right inset is the enlarged 3 cycles. (C) Detailed rise and decay time of the device under 462-nm laser irradiation (10 μW, *V*_bias_ = 4 V). (D) Comparison of response time and responsivity with other 2D material-based devices. P(VDF-TrFE), poly(vinylidene fluoride-trifluoroethylene).

The photodetector’s rise time is typically defined as the duration required for the current to ascend from 10% to 90% of its maximum value, whereas the fall time is described as the time necessary for the current to descend from 90% to 10%. As illustrated in Fig. S7, the apparatus exhibits a swift response time for pulsed illumination over a broad spectrum ranging from 365 to 1,064 nm, with a microsecond response speed in the visible range. As depicted in Fig. 3A, the response rate of the device is obviously improved after applying a bias voltage, and the optimized bias is 4 V, achieving a fast speed of ~150 μs. Furthermore, a bias voltage of only 5 mV is required to achieve the fastest response time of 26 ms under 1,064-nm irradiation (Fig. S7K), providing a solution for rapid NIR photoresponse. In Fig. 3B, the device’s response stability to light over the course of 25 light cycles is presented, and it exhibits excellent repeatable switching characteristics. When the incident power is 10 μW, as illustrated in Fig. 3C, the device has the shortest response time to a 462-nm laser, with a rise time of ≈150 μs and a fall time of ≈250 μs, which is 250 times faster than that of the MoS_2_/SiO_2_/Si photodetector (Fig. S8C). To comprehensively characterize the outstanding performance of the device, Fig. 3D emphasizes the premium performance of the device compared to previously reported devices [21,22,25,61,64–77], indicating its capability to achieve faster response speed over the broad wavelength range while maintaining a decent responsivity.

### Self-powered response of MoS_2_/LiNbO_3_ photodetector at 0 bias

**Fig. 4.**
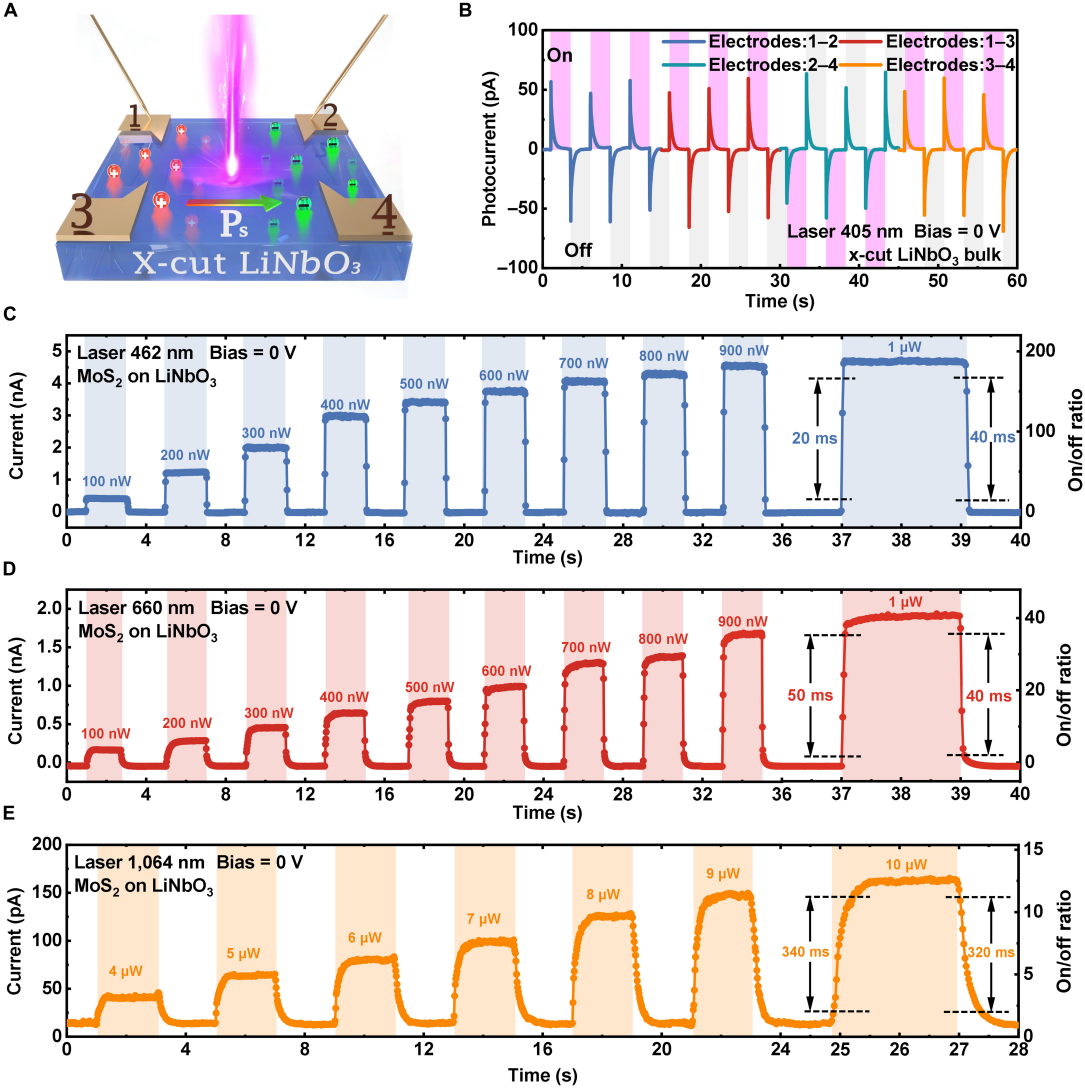
Compared characterizations of the switching behavior between pristine LiNbO_3_ and MoS_2_/ LiNbO_3_ detector. (A) Illustration of the LiNbO_3_ pyroelectric detector. (B) The relationship between photocurrent and time of the x-cut LiNbO_3_ device collected from different electrode pairs. Response time of the MoS_2_/LiNbO_3_ photodetector under (C) 462-nm, (D) 660-nm, and (E) 1,064-nm lasers (*V*_bias_ = 0 V).

The aforementioned results have clearly shown that the photodetection performance including *R*, D*, EQE, and response speed of the novel MoS_2_/LiNbO_3_ device is superior to that of the MoS_2_/SiO_2_/Si device, showing orders of magnitude improvement (Fig. S8B). To explore the reason for the remarkable improvement in device performance and reveal the interaction mechanism between LiNbO_3_ and 2D MoS_2_, we conducted systematic photoresponse measurements before and after the vdW integration process under zero bias (Fig. S9), thus obtaining the comparison between pristine LiNbO_3_ device and MoS_2_/LiNbO_3_ device (Fig. 4A). Upon a 405-nm laser irradiation, as the laser is turned on and off, forward and reverse current spikes are generated in the pristine LiNbO_3_ device (Fig. 4B), the pyroelectric current direction between electrodes 1 and 2 and 3 and 4 is the same, while the pyroelectric current direction between electrodes 1 and 3 and 2 and 4 is opposite, showing that the charges accumulated by electrodes 1 to 3 and electrodes 2 to 4 are opposite (see Fig. 4A), which is consistent with the previous results [39]. After a MoS_2_ flake is assembled onto the LiNbO_3_ substrate, the MoS_2_/LiNbO_3_ device is obtained. The light irradiation would bring in a local temperature increase in LiNbO_3_, resulting in a reduction of the spontaneous polarization intensity. Therefore, the binding ability of ferroelectric polarization on the surface charge of LiNbO_3_ is weakened, resulting in holes and electrons being injected into the MoS_2_ channel along both sides of the *z* direction, thus modulating the distribution of carriers in the channel and forming an internal electric field, driving the photogenerated carriers to move directionally, and donating the device the self-powered function without bias voltage; and the *R*, D*, and EQE of self-powered response can be found in Fig. S10. The response time of the device was evaluated under various laser wavelengths at 0 bias voltage, and the results are depicted in Fig. 4C to E. It can be seen that with the increase in incident power, the photoconductivity effect of MoS_2_ and the pyroelectric effect of LiNbO_3_ are jointly enhanced, which can boost the response speed from both aspects. In particular, the 462-nm group exhibited the fastest response time at an incident power of 1 μW, with a rise time of ≈20 ms and a fall time of ≈40 ms (Fig. 4C), further showcasing the fast response of the device in self-drive mode. Furthermore, when an externally applied bias is superimposed on the electric field generated by the pyroelectric effect, it can also facilitate the efficient separation of photogenerated electron-hole pairs and expedite the response time of the device (as shown in Fig. 3). In addition, with the aid of the pyroelectric effect of LiNbO_3_ over extraordinarily wide spectral extent and the lower dark current of MoS_2_ [45–48], the device is capable of low-noise, self-powered optical sensing in the broad range of 365 to 1,064 nm and switching ratios up to ≈190 (see Fig. 4C and Fig. S11).

### Polarization detection of MoS_2_/LiNbO_3_ photodetector

**Fig. 5.**
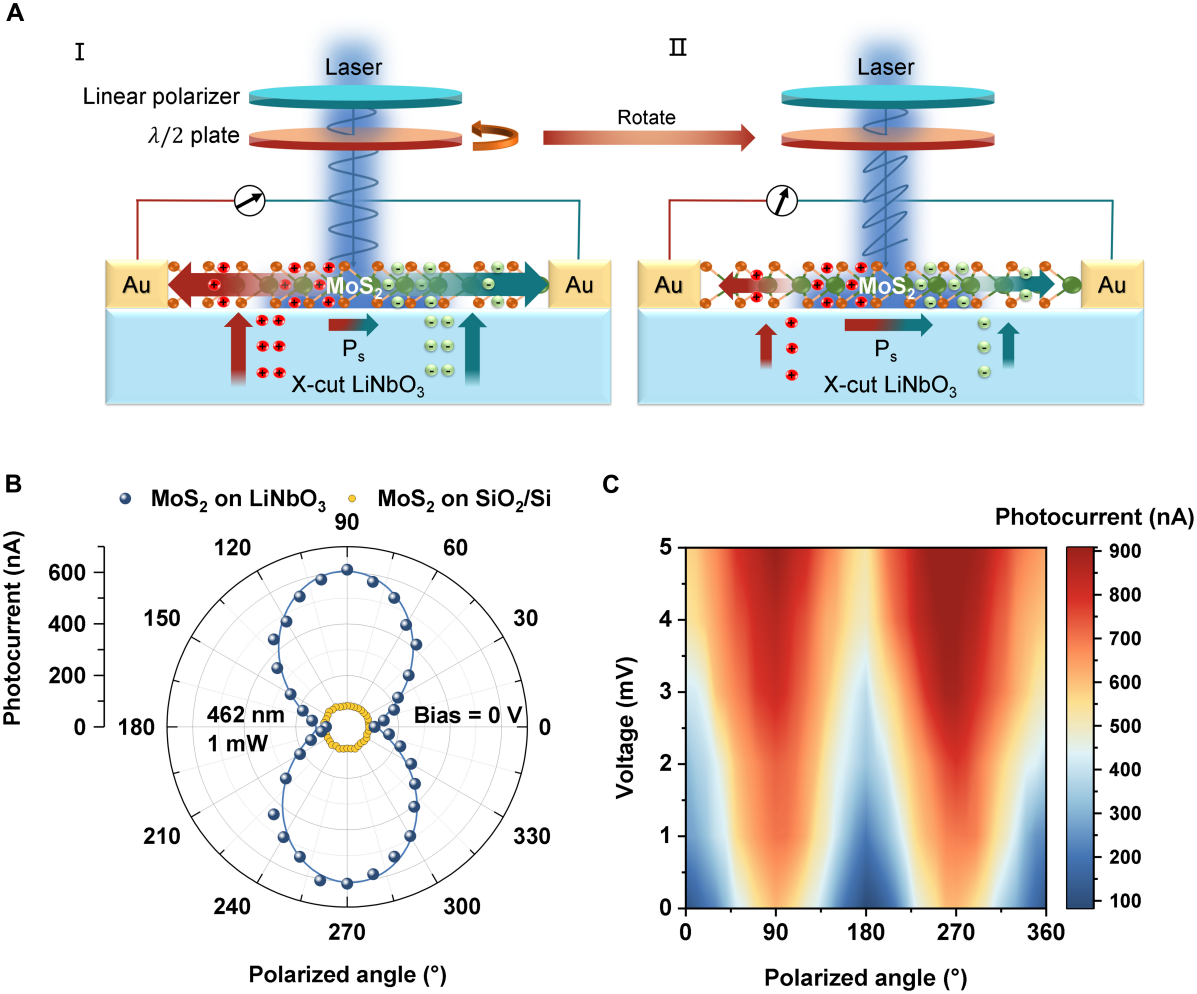
Characterization of polarization detection behavior induced by ferroelectric LiNbO_3_. (A) Schematic diagram of the setup for testing the polarization-sensitive photoresponse. From I (left) to II (right), rotating the *λ*/2 plate. (B) Variation of photocurrent with angle under different 462-nm linearly polarized lights. (C) Anisotropic response of polarization current as a function of bias voltage (*λ* = 462 nm).

The ferroelectric properties of LiNbO_3_ are mainly due to the spontaneous polarization caused by the structural anisotropy of the crystal lattice. Therefore, polarization-related characterizations become very important to reveal the working mechanism of the device. As shown in Fig. 5A, a beam of circularly polarized light is incident on MoS_2_/LiNbO_3_ device after passing through a linear polarizer, and, at the same time, the polarization angle can be continuously changed by rotating a half wave plate (more details can be found in Materials and Methods and Fig. S12). In addition to the light-absorption-induced enhancement on the carrier concentration in MoS_2_, the ferroelectric LiNbO_3_ is sensitive to polarized light because of its anisotropic crystal structure. Upon laser irradiation, a spatial separation of the charges arises in LiNbO_3_ crystal, and the resulted internal electric field would lead to a modulation in the refractive indices [49,50], such photorefractive effect enables the polarization sensitive characteristic for the anisotropic LiNbO_3_ [51–53], and the polarization-angle-dependent pyroelectric charges would inject into the MoS_2_ channel (more details about the band structures of MoS_2_ and LiNbO_3_ can be found in Fig. S13) [78–80], thus enabling the polarization detection ability to the MoS_2_/LiNbO_3_ detector, while such phenomenon cannot be observed in MoS_2_/SiO_2_/Si detector (see Fig. S8D). Then, the output current of the detector is collected by the source meter to obtain the polarization response characteristics of the device. Figure 5B presents the relationship between photocurrent and polarized angle, with the MoS_2_/LiNbO_3_ detector showing a typical “8” shape, indicating a good polarization-sensitive behavior; more importantly, the photocurrent’s polarization dichroic ratio attains the maximum value of 7.42 when the bias voltage is 0, and similar behavior is also observed at other wavelengths (Fig. S14). However, the result collected from a MoS_2_/SiO_2_/Si detector is different, showing that the photocurrent is independent of the polarization angle, which can be ascribed to the isotropy of both MoS_2_ lattice [28,29] and SiO_2_/Si substrate. Figure 5C displays a contour plot depicting the output photocurrent as a function of the incident polarization direction of the 462-nm laser and the bias voltage, from which we can see that the polarization dichroic ratio of the photocurrent gradually decreases as the bias voltage increases, because a larger bias voltage could drive a larger current in the channel, while the pyroelectric current from LiNbO_3_ injected into the MoS_2_ channel is mainly controlled by the laser irradiation and fixed; thus, its proportion to the total current decreases, resulting in a reduced dichroic ratio.

## Conclusion

In summary, a MoS_2_/LiNbO_3_ heterojunction photodetector is designed and constructed, in which the ferroelectric characteristics of LiNbO_3_ and the semiconductor characteristics of MoS2 are well integrated and utilized, leading to improved photodetection performance: self-powered operation, broad detection range of 365 to 1,064 nm, fast rise/fall time (≈150 μs/250 μs), large switching ratio (≈190), high responsivity (17.3 A·W^−1^), high specific detectivity (4.3 × 10^11^ Jones), and large EQE (4645.78%). More interestingly, the internal electric field of LiNbO_3_ can be regulated by polarized irradiation, thus producing a dichroic ratio of 7.42 for the MoS_2_/LiNbO_3_ detector in self-powered mode, compared favorably than most MoS_2_-based heterostructured devices. On the basis of the foregoing results, this work has greatly promoted the investigation to elucidate the contribution of ferroelectrics in augmenting the photodetection performance, proposing a feasible strategy for cost-effective and high-performance broadband polarization-sensitive photodetectors.

## Materials and Methods

### Device fabrication

The MoS_2_/LiNbO_3_ device was fabricated through a vdW integration approach that combined a standard photolithography process and dry transfer process. A 500-μm-thick x-cut LiNbO_3_ was utilized as the substrate (1.2 cm × 1 cm), and photolithography was used to define the device pattern onto the substrate, followed by the sequential deposition of a Cr layer (5 nm) and an Au layer (80 nm), forming the electrodes for electric contact. Afterward, the MoS_2_ thin layer was exfoliated by using Scotch tape, then picked up by a polydimethylsiloxane film (Metatest Corporation), and attached to a transparent glass slide. After that, the target MoS_2_ sample was precisely positioned above the LiNbO_3_ electrode under the transfer station (Metatest Corporation, E1-T), and then the sample was lowered and gradually contacted to the prefabricated electrodes; after they are fully touched, the specimen was heated up to 80 °C for 10 min, and then the polydimethylsiloxane can be removed and finally obtained the MoS_2_/LiNbO_3_ device. It is worth noting that no solution was introduced during the whole process, minimizing the damage to the sample during the processing, and more details can be found in Fig. S1.

### Characterizations

The Bioscope Catalyst/Multimode scanning probe microscope was utilized to measure the thickness of MoS_2_ and Au electrodes. The LabRAM INV laser micro-confocal Raman spectrometer characterized the lattice vibration behavior of the MoS_2_/LiNbO_3_ heterojunction using a 532-nm laser. A schematic of the system used to test the optoelectronic performance of the MoS_2_/LiNbO_3_ photodetector is displayed in Fig. S15. A Tektronix AFG3102 signal generator was used to modulate lasers of varying wavelengths, intermittently illuminating the device. The Keithley 2601B source meter was used to collect the device’s volt–ampere and time–ampere characteristic curves under different laser irradiation conditions. Specifically, a linear polarizer and a half-wave plate were introduced into the optical path, enabling us to characterize the polarization-sensitive photodetection behavior (Fig. S12). The power of the laser was measured by the NOVA II OPHIR optical power meter, all the device measurements were conducted in the atmosphere at room temperature, and all device data were recorded by the Keithley KickStart software.

### Band structure calculation

The band structures of MoS_2_ and LiNbO_3_ were calculated on the basis of the density functional theory, as implemented in the Vienna ab initio package, and Perdew–Burke–Ernzerhof method was used for the exchange–correlation functional [81–83]. The cutoff energy was 600 eV, and all the atoms in the slab were allowed to fully relax until the forces acting on them are less than 0.01 eV·Å^−1^.

## Acknowledgments

We gratefully acknowledge the computer time provided by the Chinese Academy of Sciences Shanghai Institute of Silicate for the band structure calculation. **Funding:** This work was funded in part by the NSAF (U2030103,U2230111), the National Natural Science Foundation of China (61775084, 62075088, and 12005210), the Youth Talent Support Programme of Guangdong Provincial Association for Science and Technology (SKXRC202304), the Natural Science Foundation of Guangdong Province (2020A1515010791, 2021A0505030036, and 2022A1515110970), the Fundamental and application foundation project of Guangzhou (202201010654), and the Fundamental Research Funds for the Central Universities (21622107 and 21622403). **Author contributions:** H.G., T.Y., and H.L. supervised the project, conceived, and designed the experiments. Z.H. fabricated the device and performed the measurements with the help of X.L., J.C., M.X., K.L., R.A., L.M., and F.M., and all the authors contributed to the data analysis and discussions. Z.H., H.G., T.Y., and H.L. wrote the manuscript. All authors contributed extensively to the work presented in this paper, and the authors read and approved the final manuscript. **Competing interests:** The authors declare that they have no competing interests.

## Data Availability

Supplementary information is available in the online version of the paper.

## Supplementary Materials

Supplementary 1 Fig. S1. The fabrication process of the vdW integration method for constructing MoS_2_/LiNbO_3_ heterostructured device.
Fig. S2. Detailed microstructure of the MoS_2_/LiNbO_3_ photodetector.
Fig. S3. Scanning electron microscopy image and energy-dispersive spectroscopy mapping of the MoS_2_/LiNbO_3_ photodetector.
Fig. S4. *IV* curves of the MoS_2_/LiNbO_3_ photodetector illuminated by different lasers.
Fig. S5. Wavelength dependence of the MoS_2_/LiNbO_3_ photodetector.
Fig. S6. Photodetection performance of the MoS_2_/LiNbO_3_ device at 808-nm laser irradiation.
Fig. S7. Detailed rise and decay times of the MoS_2_/LiNbO_3_ photodetector under different laser irradiation.
Fig. S8. Detailed photodetection characterizations of the MoS_2_ device fabricating on SiO_2_/Si substrate.
Fig. S9. Pyroelectric behavior of the pristine LiNbO_3_ device.
Fig. S10. Self-powered response performance of the MoS_2/_LiNbO_3_ photodetector.
Fig. S11. Photoswitching stability characteristics of the MoS_2_/LiNbO_3_ photodetector.
Fig. S12. Schematic of the polarized light detection system.
Fig. S13. Band structure diagram at the interface of MoS_2_ and LiNbO_3_.
Fig. S14. Detailed polarization dependent photodetection results.
Fig. S15. Schematic of the photodetection test setup.
Table S1. The *α* value and coefficient of determination of the fitted curves.
