# Supplementary material for: Broadband, Polarization-Sensitive, and Self-Powered High-Performance Photodetection of Hetero-Integrated MoS2 on Lithium Niobate
Source: Research (Wash D C). 2023 Jul 20;6:0199. doi: 10.34133/research.0199 (PMC10357351; doi:10.34133/research.0199)
Supplement: Supplementary 1 — Fig. S1. The fabrication process of the vdW integration method for constructing MoS2/LiNbO3 heterostructured device. Fig. S2. Detailed microstructure of the MoS2/LiNbO3 photodetector. Fig. S3. Scanning electron microscopy image and energy-dispersive spectroscopy mapping of the MoS2/LiNbO3 photodetector. Fig. S4. IV curves of the MoS2/LiNbO3 photodetector illuminated by different lasers. Fig. S5. Wavelength dependence of the MoS2/LiNbO3 photodetector. Fig. S6. Photodetection performance of the MoS2/LiNbO3 device at 808-nm laser irradiation. Fig. S7. Detailed rise and decay times of the MoS2/LiNbO3 photodetector under different laser irradiation. Fig. S8. Detailed photodetection characterizations of the MoS2 device fabricating on SiO2/Si substrate. Fig. S9. Pyroelectric behavior of the pristine LiNbO3 device. Fig. S10. Self-powered response performance of the MoS2/LiNbO3 photodetector. Fig. S11. Photoswitching stability characteristics of the MoS2/LiNbO3 photodetector. Fig. S12. Schematic of the polarized light detection system. Fig. S13. Band structure diagram at the interface of MoS2 and LiNbO3. Fig. S14. Detailed polarization dependent photodetection results. Fig. S15. Schematic of the photodetection test setup. Table S1. The α value and coefficient of determination of the fitted curves. [file research.0199.f1.docx]

**Supplementary Materials**

**Broadband, Polarization-Sensitive and Self-Powered High-Performance Photodetection of Hetero-Integrated MoS_2_ on Lithium Niobate**

Zhigang He^1^, Heyuan Guan^1,*^, Xijie Liang^1^, Junteng Chen^1,2^, Manyan Xie^1,2^, Kaiwen Luo^1,2^, Ran An^3^, Liang Ma^4^, Fengkai Ma^1^, Tiefeng Yang^1,*^, Huihui Lu^1,2*^

*1Guangdong Provincial Key Laboratory of Optical Fiber Sensing and Communications, Jinan University, Guangzhou 510632, China*

*2Key Laboratory of Optoelectronic Information and Sensing Technologies of Guangdong Higher Education Institutes, Jinan University, Guangzhou 510632, China*

*3Institute of Fluid Physics, China Academy of Engineering Physics, Mianyang 621900, China*

*^4^College of Physics and Electronic Engineering, Hengyang Normal University, Hengyang 421008, China*

*Correspondence: [ttguanheyuan@jnu.edu.cn](mailto:ttguanheyuan@jnu.edu.cn); [yangtiefeng2022@jnu.edu.cn](mailto:yangtiefeng2022@jnu.edu.cn); [thuihuilu@jnu.edu.cn](mailto:thuihuilu@jnu.edu.cn)


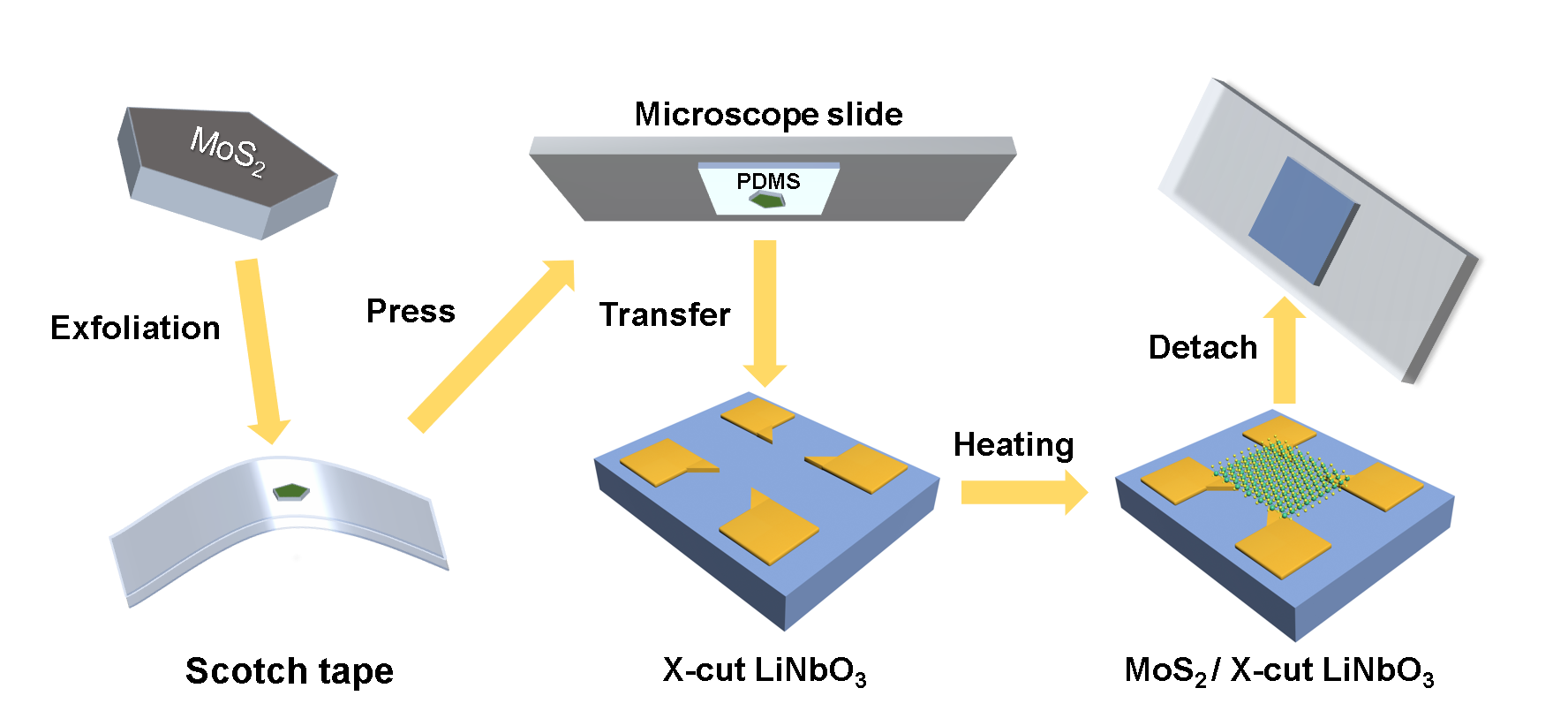


**Figure S1:** The fabrication process of the van der Waals integration method for constructing MoS_2_/LiNbO_3_ heterostructured device.


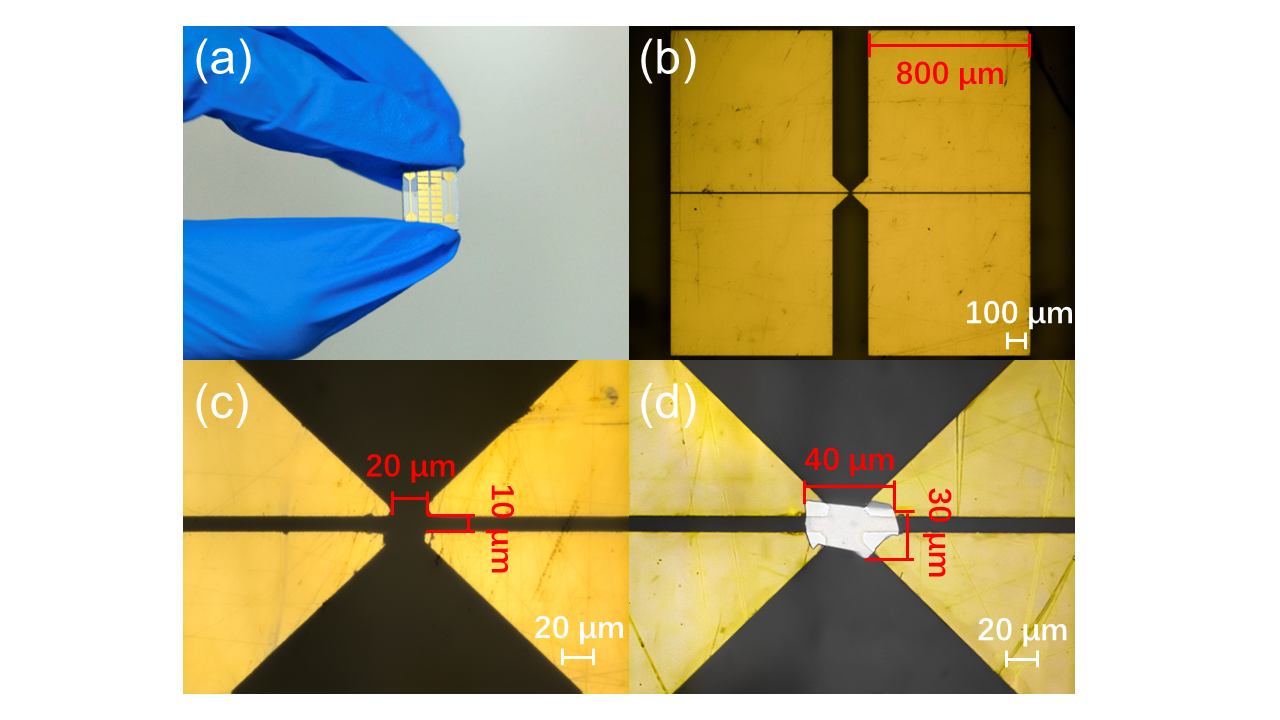


**Figure S2:** (a) Intuitive appearance of a LiNbO_3_ chip covered with Au electrodes. (b) Optical microscope image of the overall shape of a group of Au electrodes on LiNbO_3_ substrate, consisting of rectangular part with lateral size of ≈ 800 µm × 800 µm (for contact with the probes) and the central triangular protrusions (for contact with 2D materials). (c) The interval between the triangular protrusions of Au electrodes. (d) The specific dimensions of the 2D-MoS_2_ used in the MoS_2_/LiNbO_3_ heterojunction photodetector.


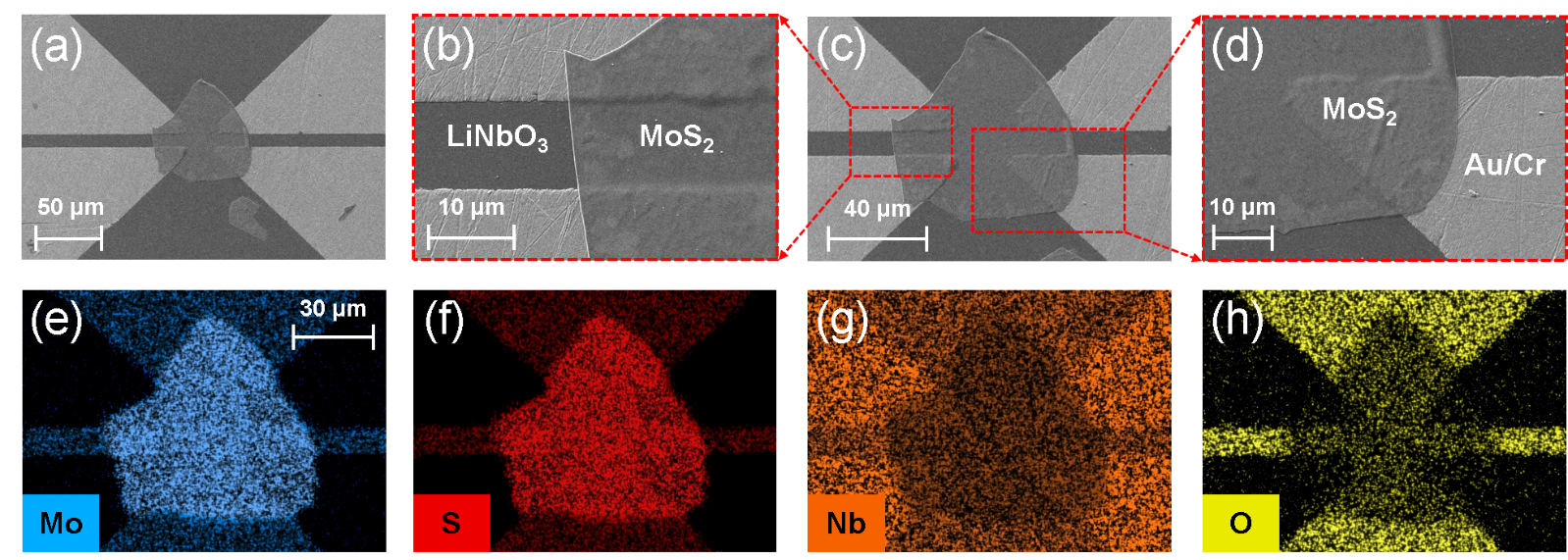


**Figure S3:** (a) SEM image of the MoS_2_/LiNbO_3_ heterojunction photodetector. (b) LiNbO_3_ covered with multilayer MoS_2_. (c) SEM image with a higher magnification level. (d) Au electrode covered with multilayer MoS_2_. Image of the device with EDS mapping showing the presence of (e) Mo, (f) S, (g) Nb, and (h) O elements.


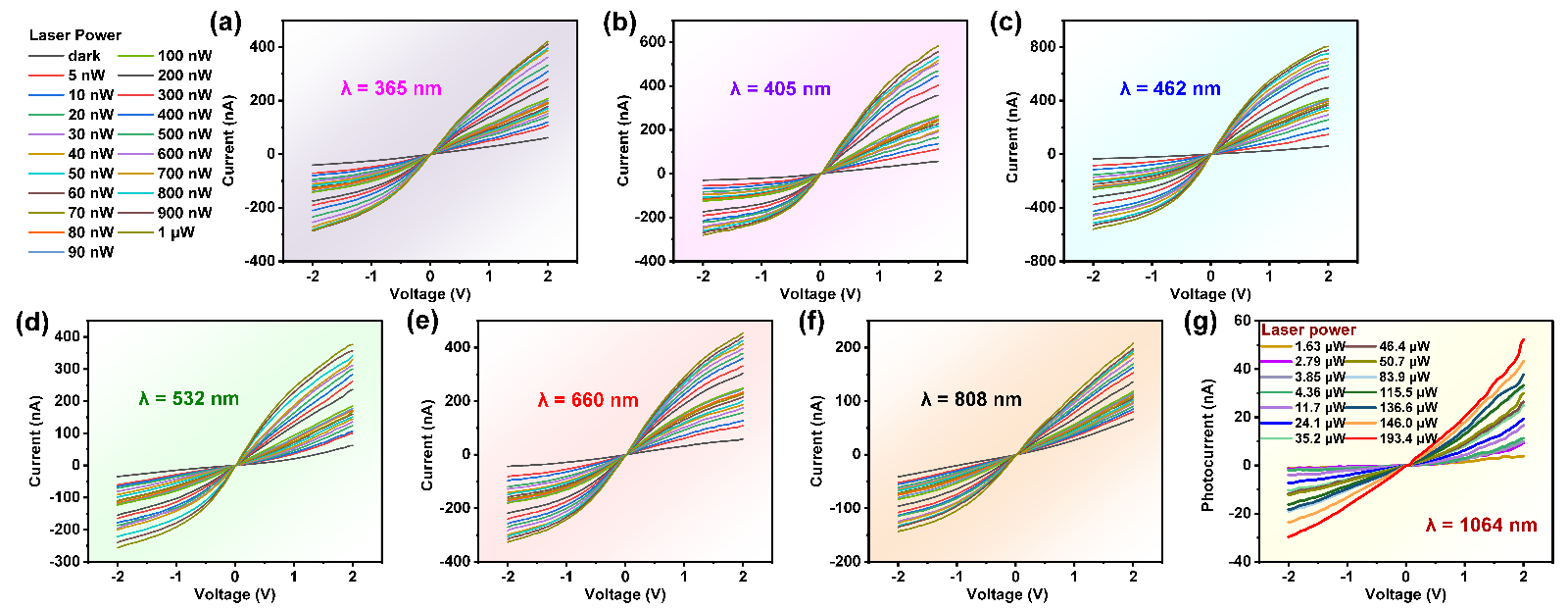


**Figure S4:** IV curves of the MoS_2_/LiNbO_3_ photodetector illuminated by different lasers. (a) 365 nm, (b) 405 nm, (c) 462 nm, (d) 532 nm, (e) 660 nm, (f) 808 nm, and (g) 1064 nm lasers.


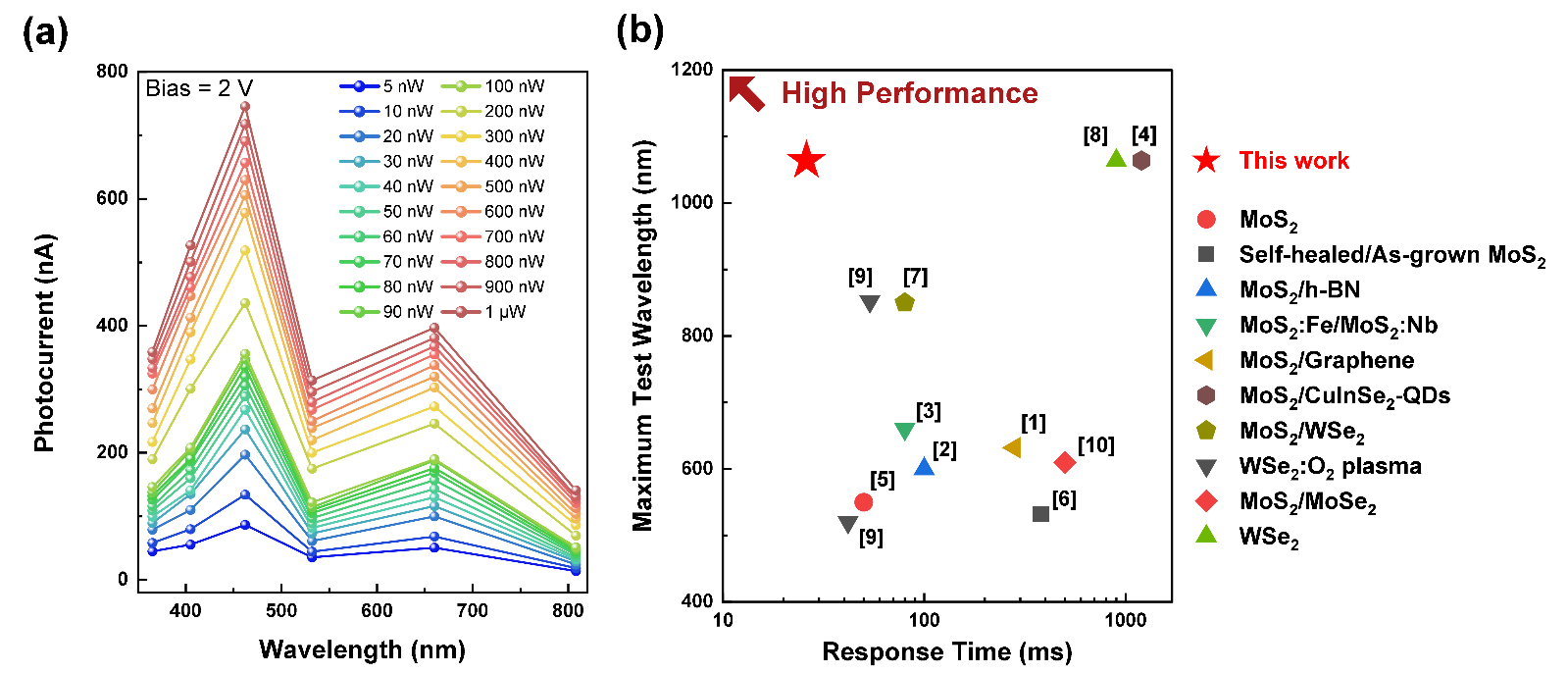


**Figure S5:** (a) The photocurrent of the MoS_2_/LiNbO_3_ heterojunction photodetector in the wavelength range of 365–808 nm. (b) Maximum test wavelength and response time of the MoS_2_/LiNbO_3_ heterojunction photodetector compared to the reported photodetectors.


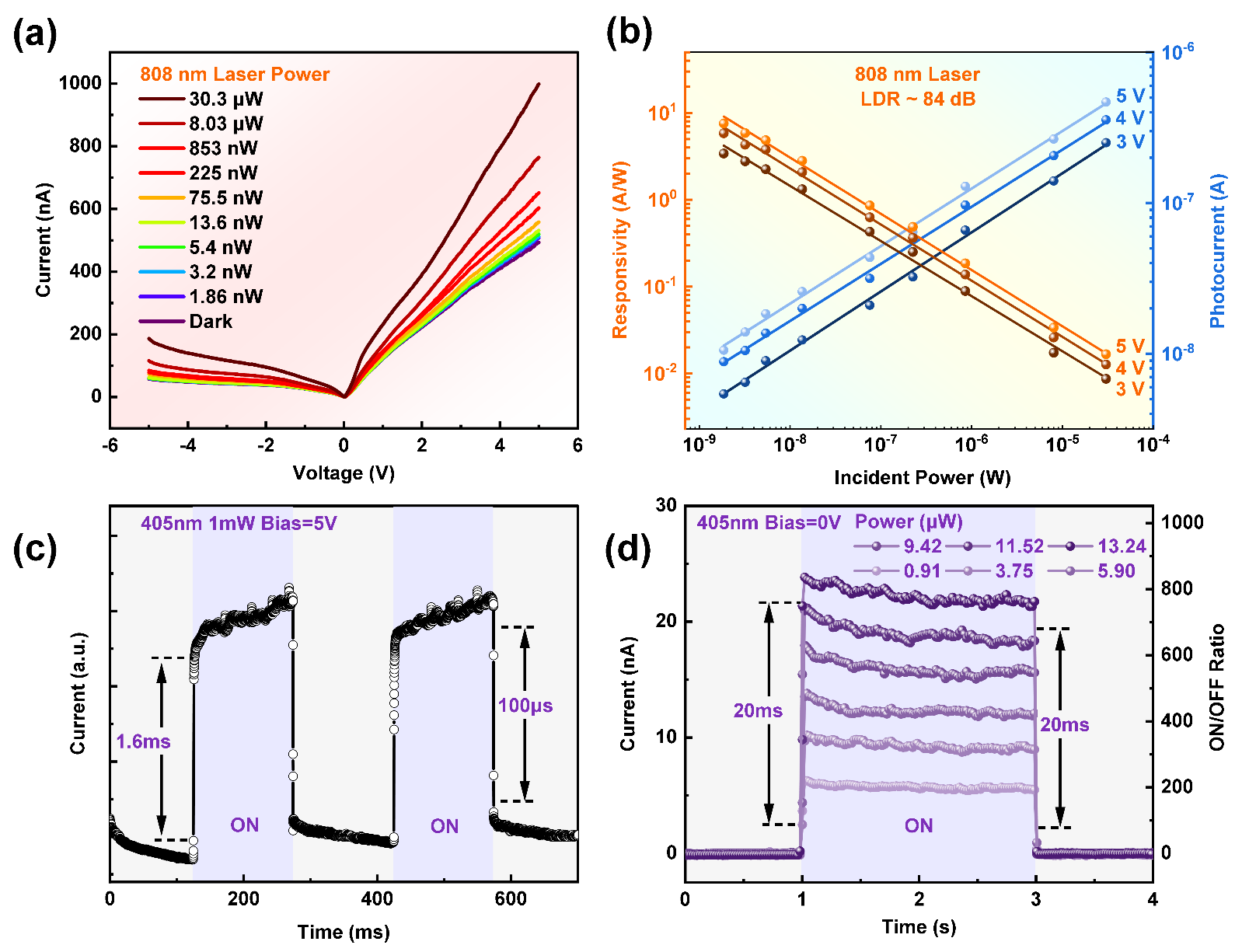


**Figure S6:** Photodetection performance of the MoS_2_/LiNbO_3_ device at 808 nm laser irradiation. (a) IV curves of the MoS_2_/LiNbO_3_ photodetector under 808 nm irradiation at different incident powers. (b) Photocurrent, responsivity, and linear dynamic range of the device under 808 nm irradiation.


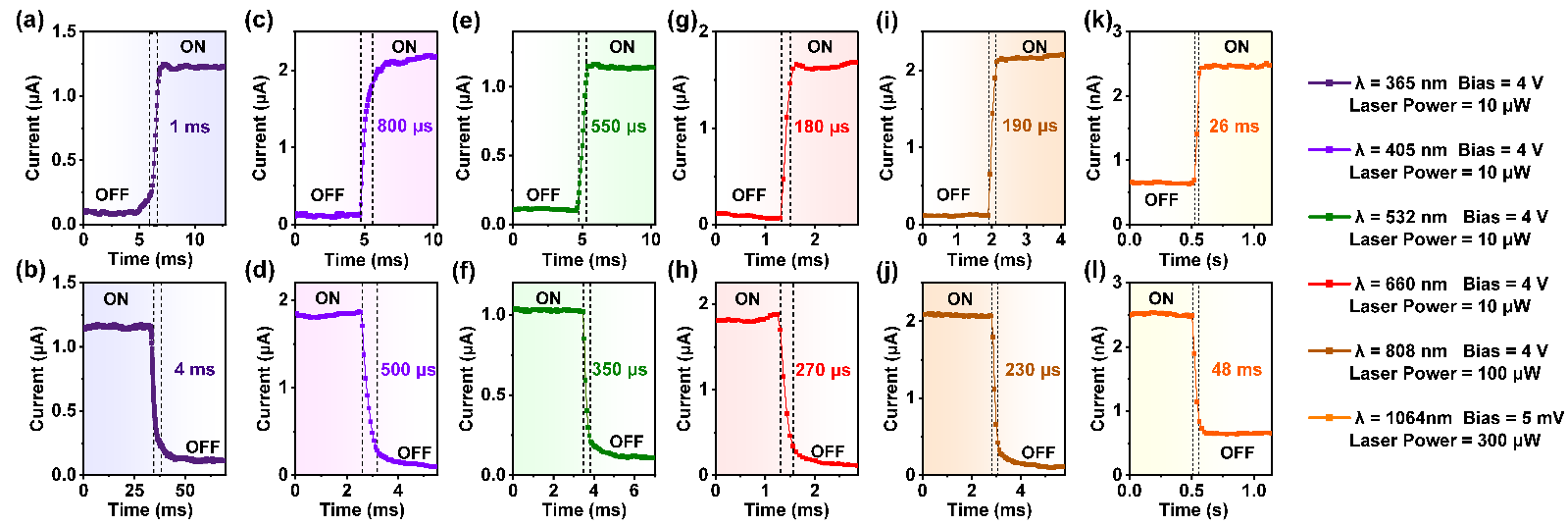


**Figure S7:** Detailed rise and decay time of the MoS_2_/LiNbO_3_ photodetector under (a, b) 365, (c, d) 405, (e, f) 532, (g, h) 660, (i, j) 808, and (k, l) 1064 nm lasers, respectively (V_bias_ = 4 V).


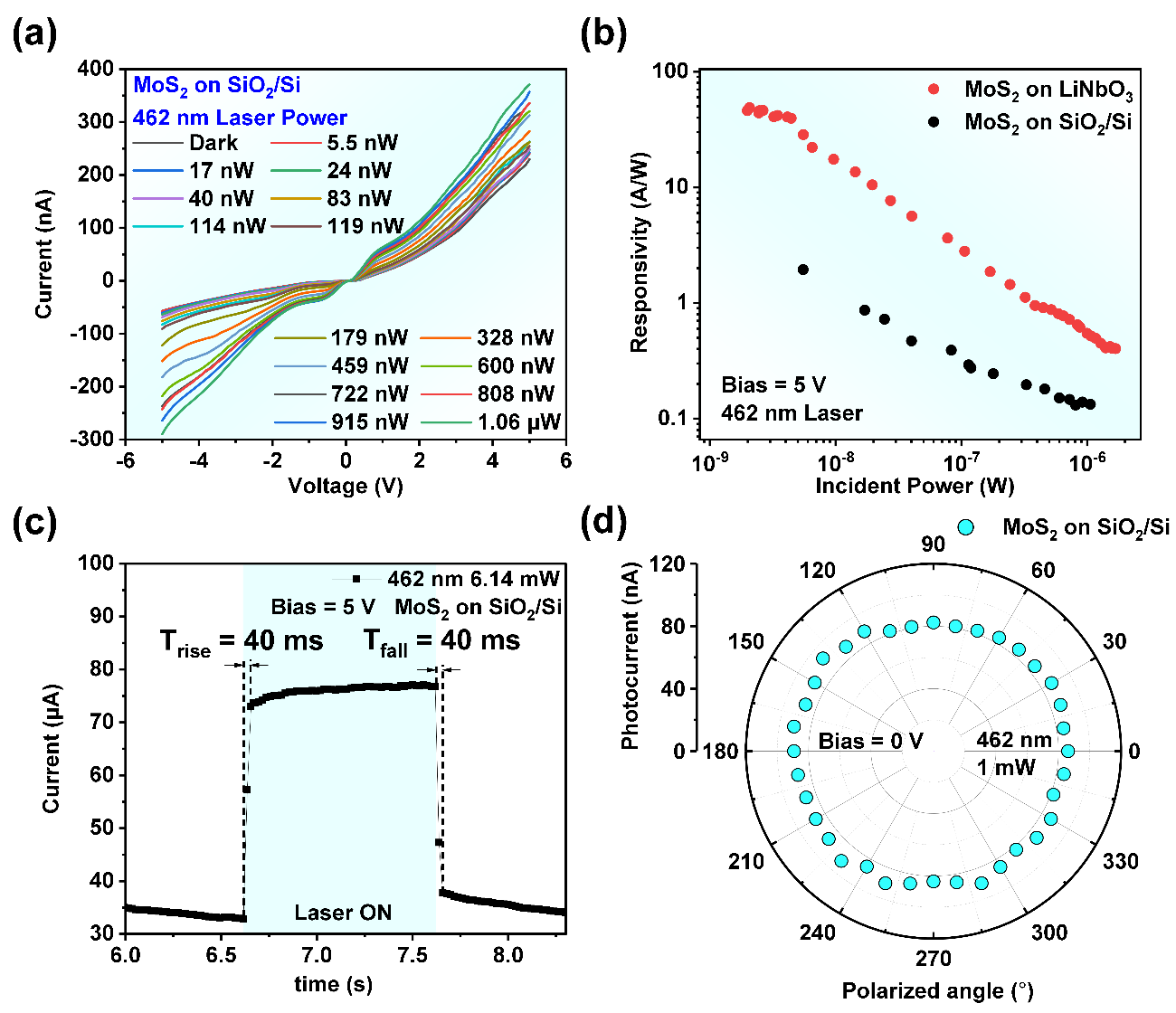


**Figure S8:** Detailed photodetection characterizations of the MoS_2_ device fabricating on SiO_2_/Si substrate. (a) IV curves of the MoS_2_/SiO_2_/Si photodetector under different laser powers. (b) Comparison of responsivity of the MoS_2_/LiNbO_3_ and MoS_2_/SiO_2_/Si photodetector under 462 nm laser (V_bias_ = 5 V). (c) Response time of the MoS_2_/SiO_2_/Si photodetector under 462 nm laser (V_bias_ = 5 V). (d) Photocurrent of the MoS_2_/SiO_2_/Si photodetector under different 462 nm linearly polarized light (V_bias_ = 0 V).


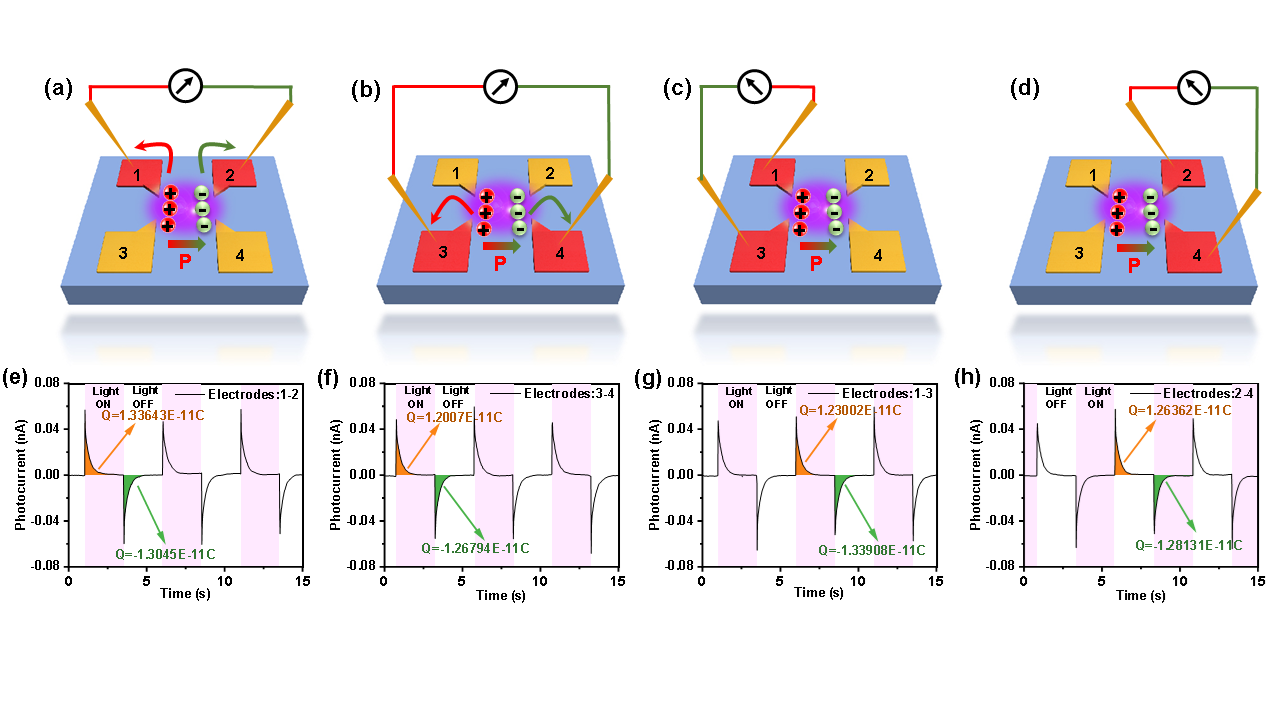


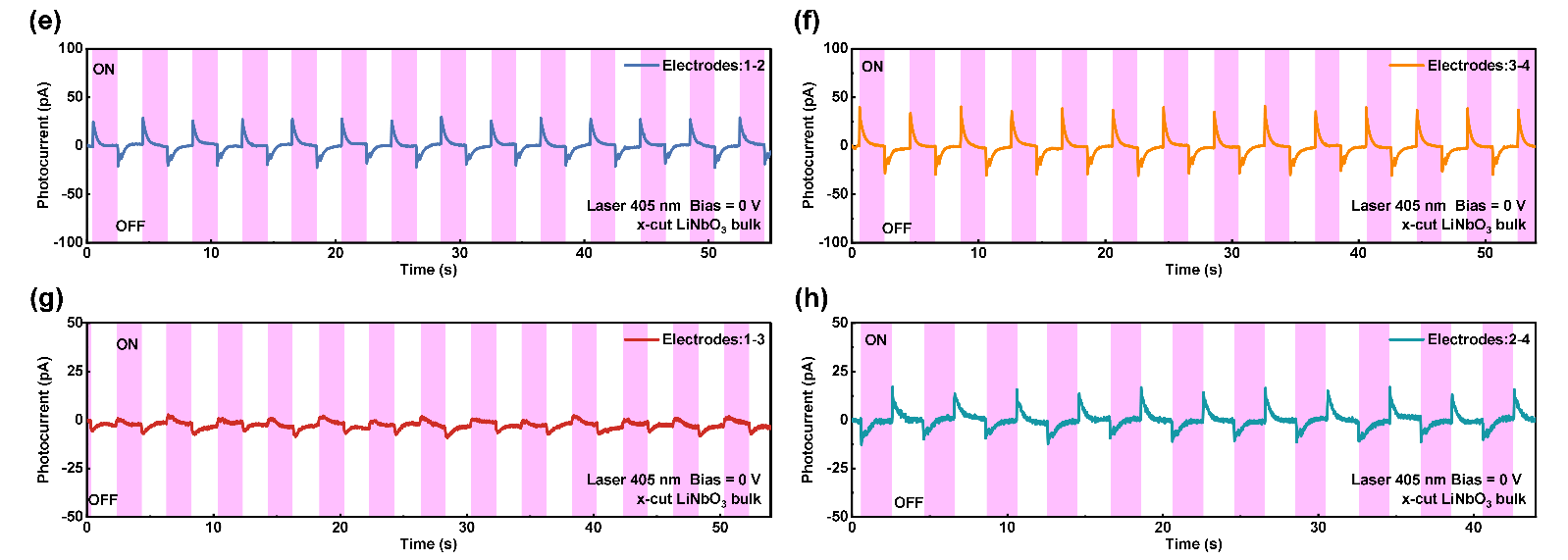


**Figure S9:** Schematic diagram of pyroelectric effect measurement of LiNbO_3_ pyroelectric detector at (a) 1-2 electrodes, (b) 3-4 electrodes, (c) 1-3 electrodes, and (d) 2-4 electrodes, respectively. Pyroelectric current of LiNbO_3_ pyroelectric detector at (e) 1-2 electrodes, (f) 3-4 electrodes, (g) 1-3 electrodes, and (h) 2-4 electrodes, respectively (*P*_in_ = 1 mW).


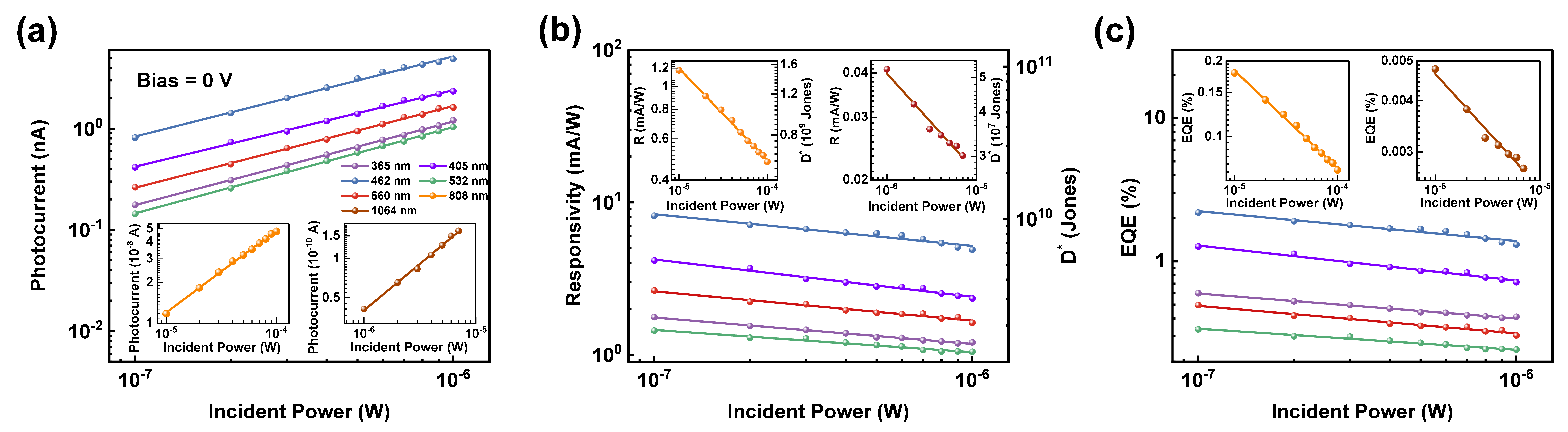


**Figure S10:** (a) Extracted photocurrent as a function of incident power (*V*_bias_ = 0 V). (b) Comparison of *D** and *R* under different laser irradiation (*V*_bias_ = 0 V). (c) Comparison of *EQE* under different laser irradiation (*V*_bias_ = 0 V).


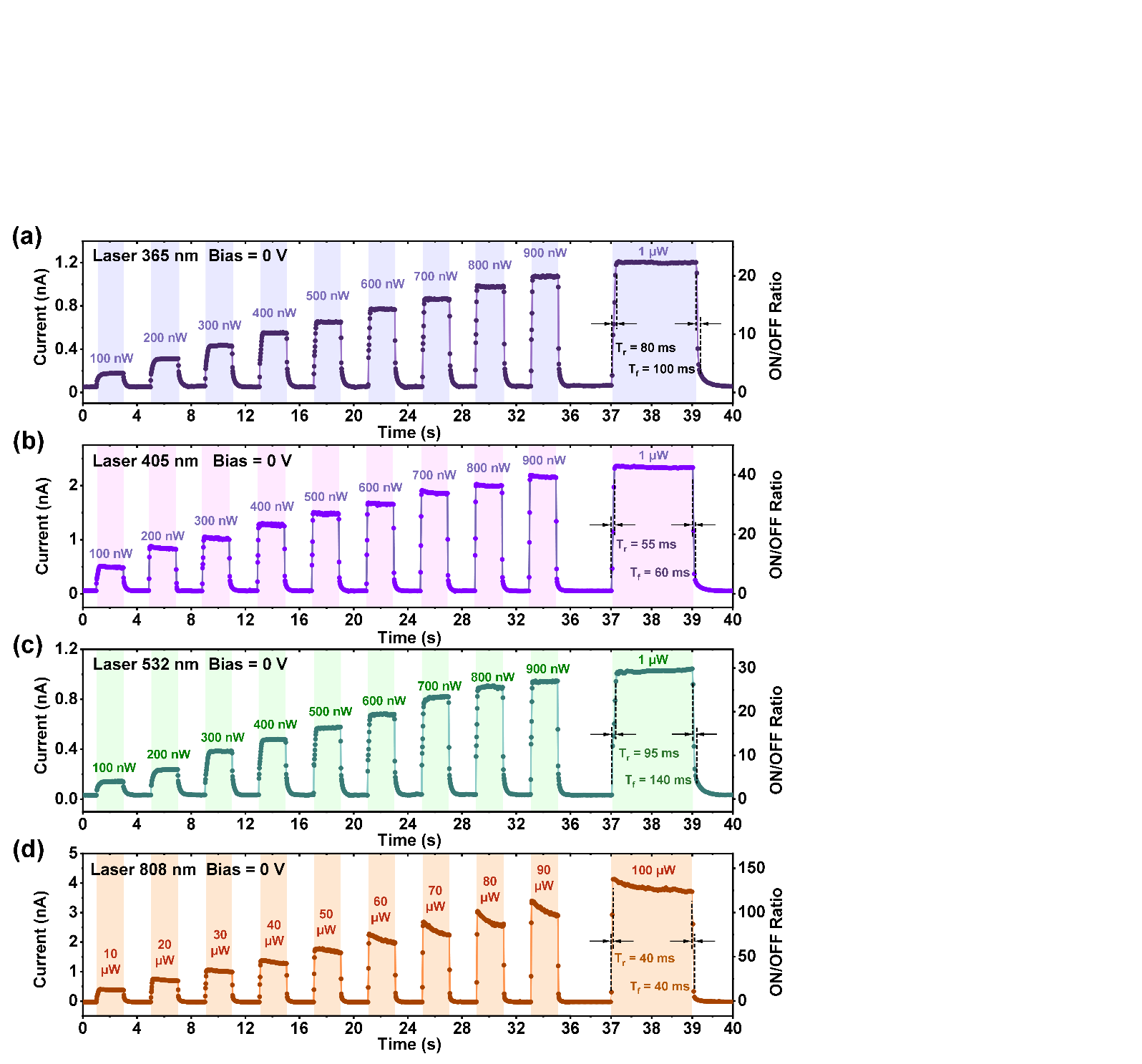


**Figure S11:** Photoswitching stability characteristics of the MoS_2_/LiNbO_3_ photodetector under (a) 365, (b) 405, (c) 532, and (d) 808 nm lasers, respectively (*V*_bias_ = 0 V).


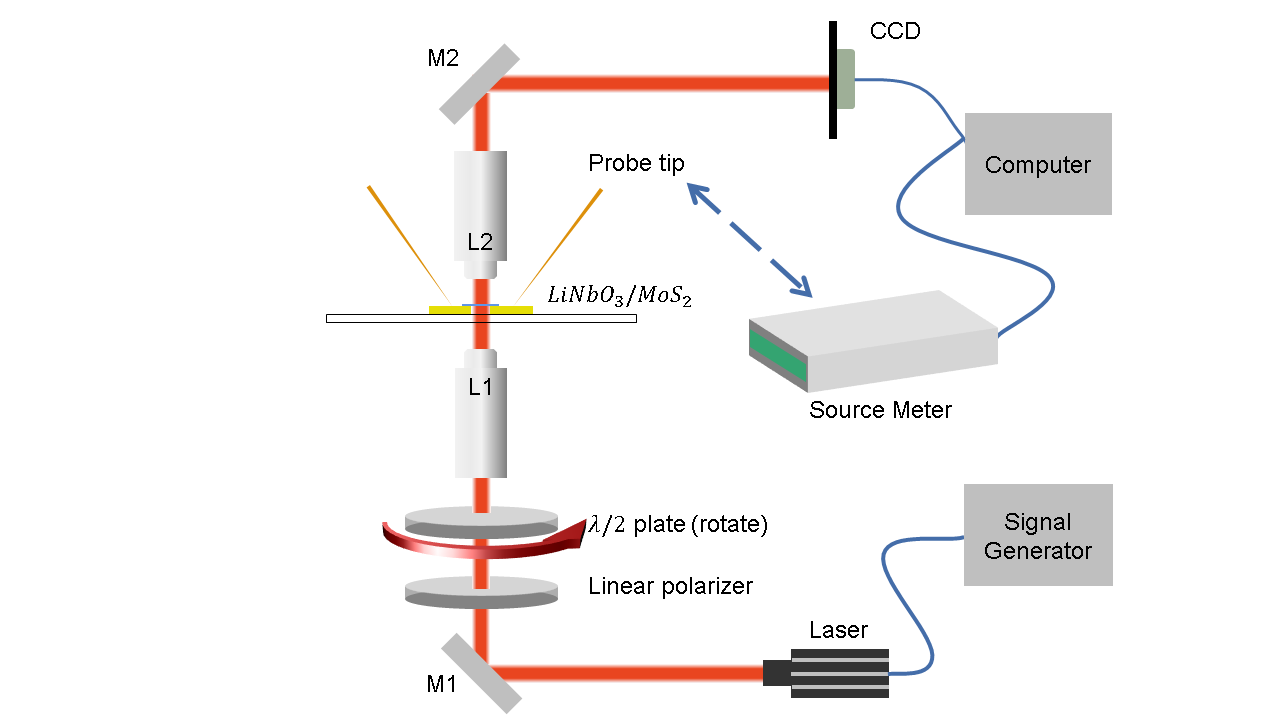


**Figure S12:** Schematic of the polarized light detection system.


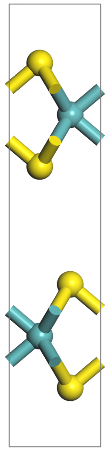

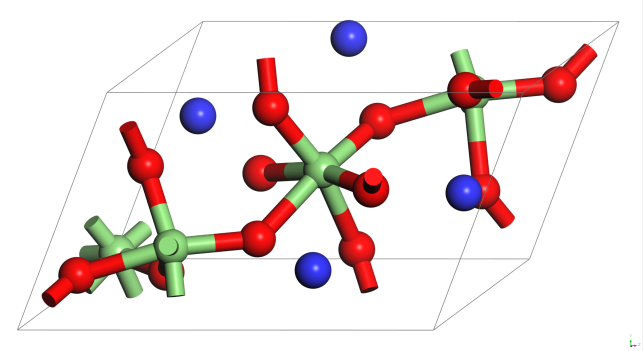

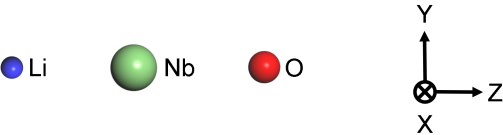

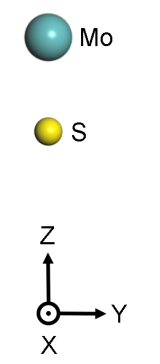

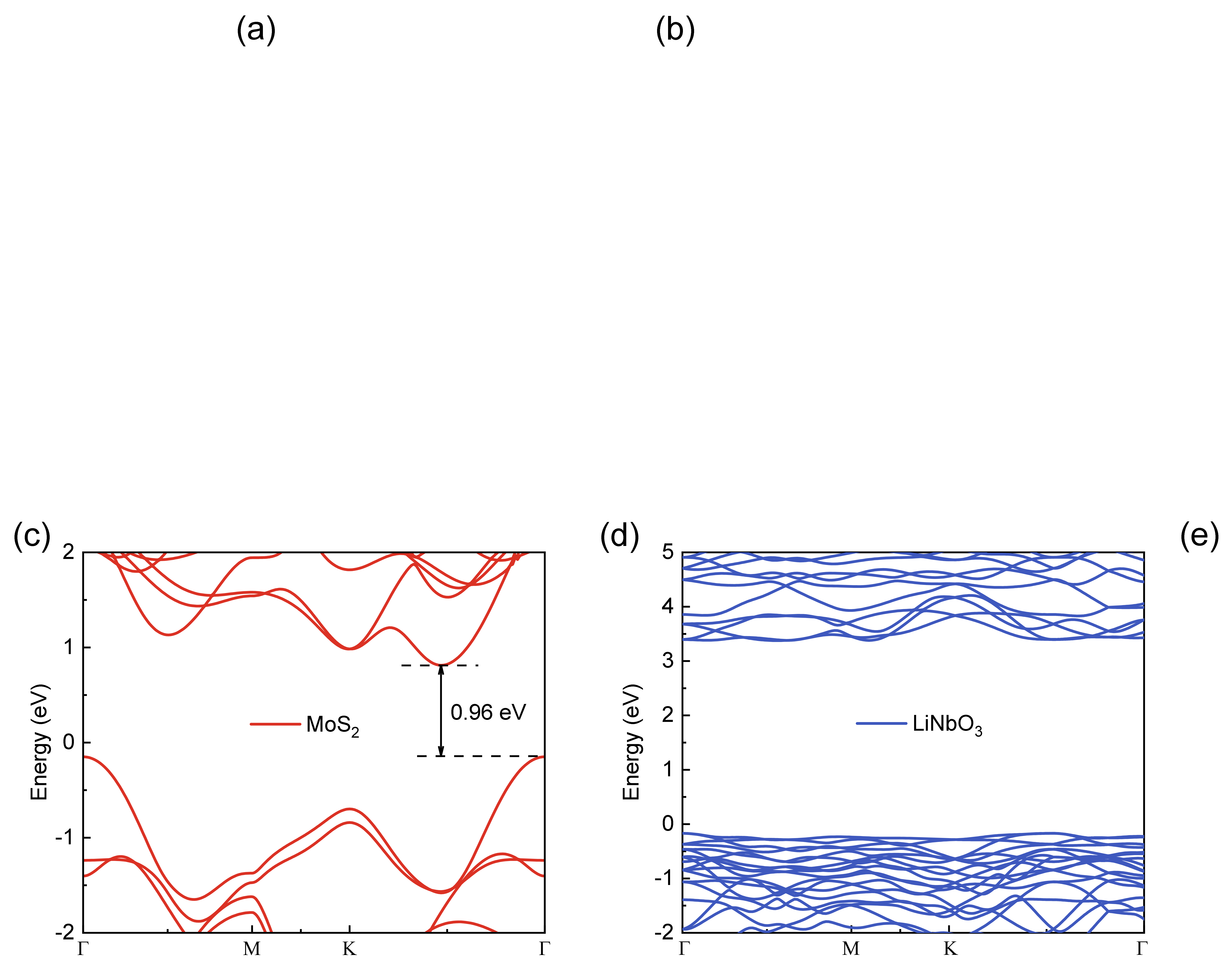

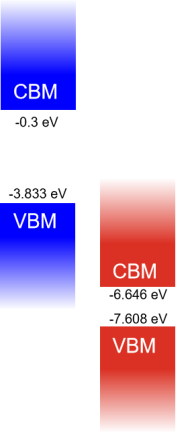


**Figure S13:** (a) MoS_2_ structure used for calculation. (b) LiNbO_3_ structure used for calculation. (c) Calculated band structure of MoS_2_. (d) Calculated band structure of LiNbO_3_. (e) Band structure diagram at the interface of MoS_2_ and LiNbO_3_.


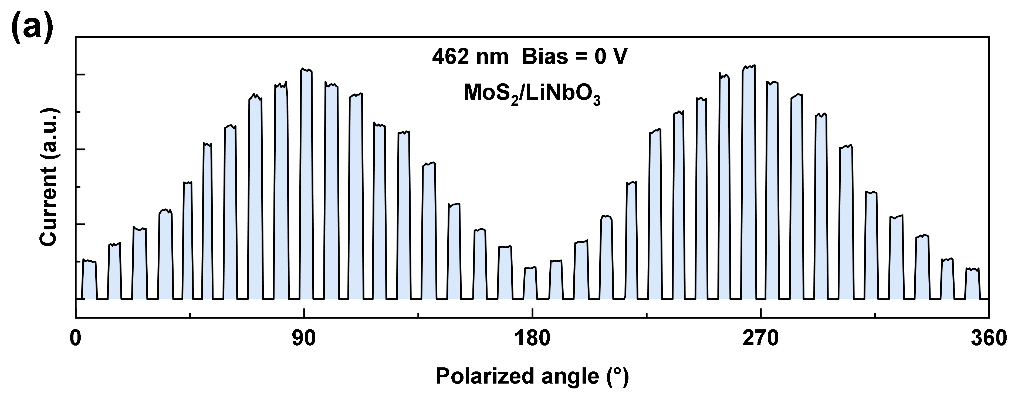


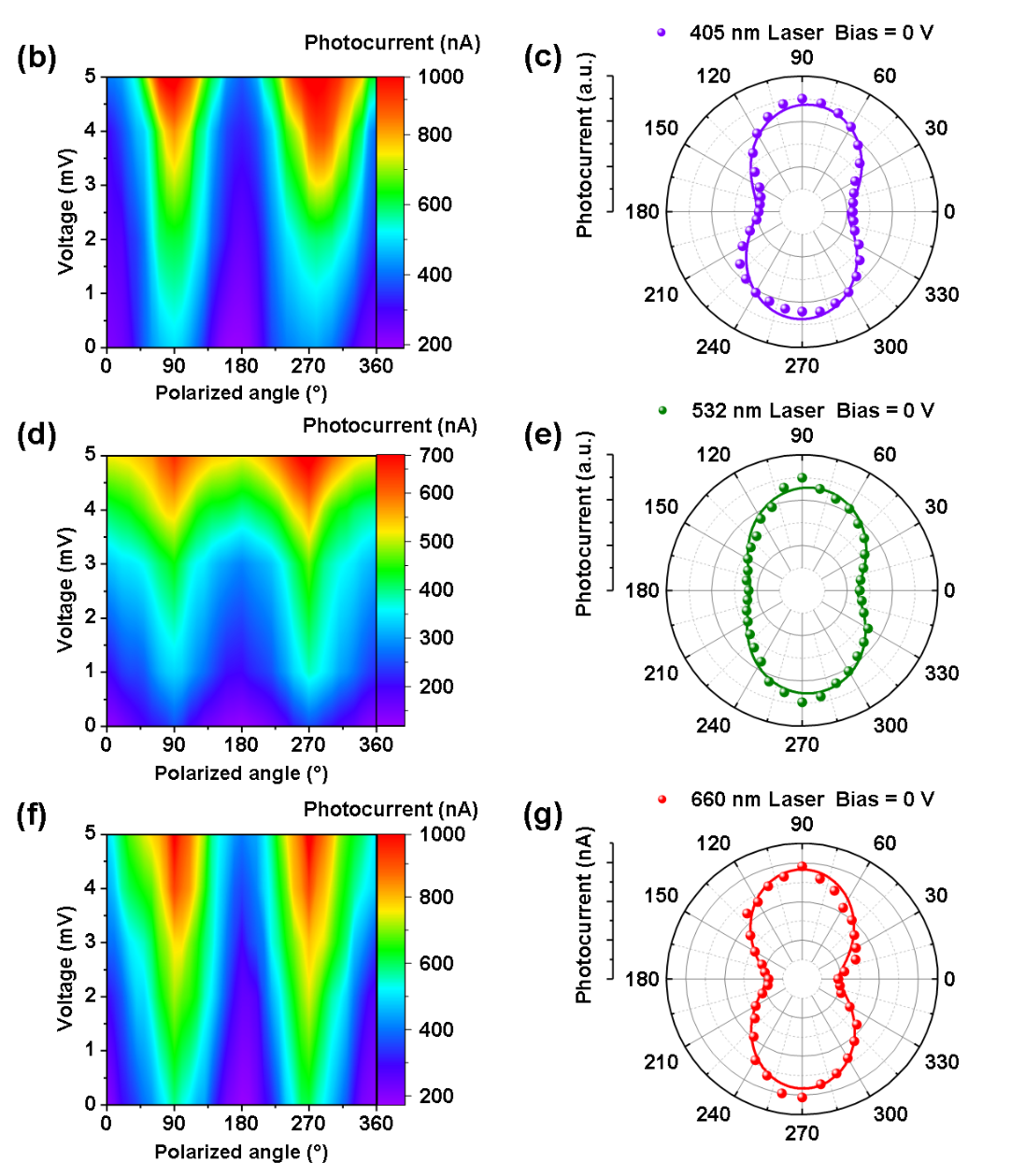


**Figure S14:** Detailed polarization dependent photodetection results. (a) Polarized light response test method. The anisotropic response of photocurrent under (b) 405, (d) 532, and (f) 660 nm laser irradiation, respectively, is a function of the bias voltage. Under (c) 405, (e) 532, and (g) 660 nm laser irradiation, respectively, the photocurrent of the device changes with the linear polarization angle.


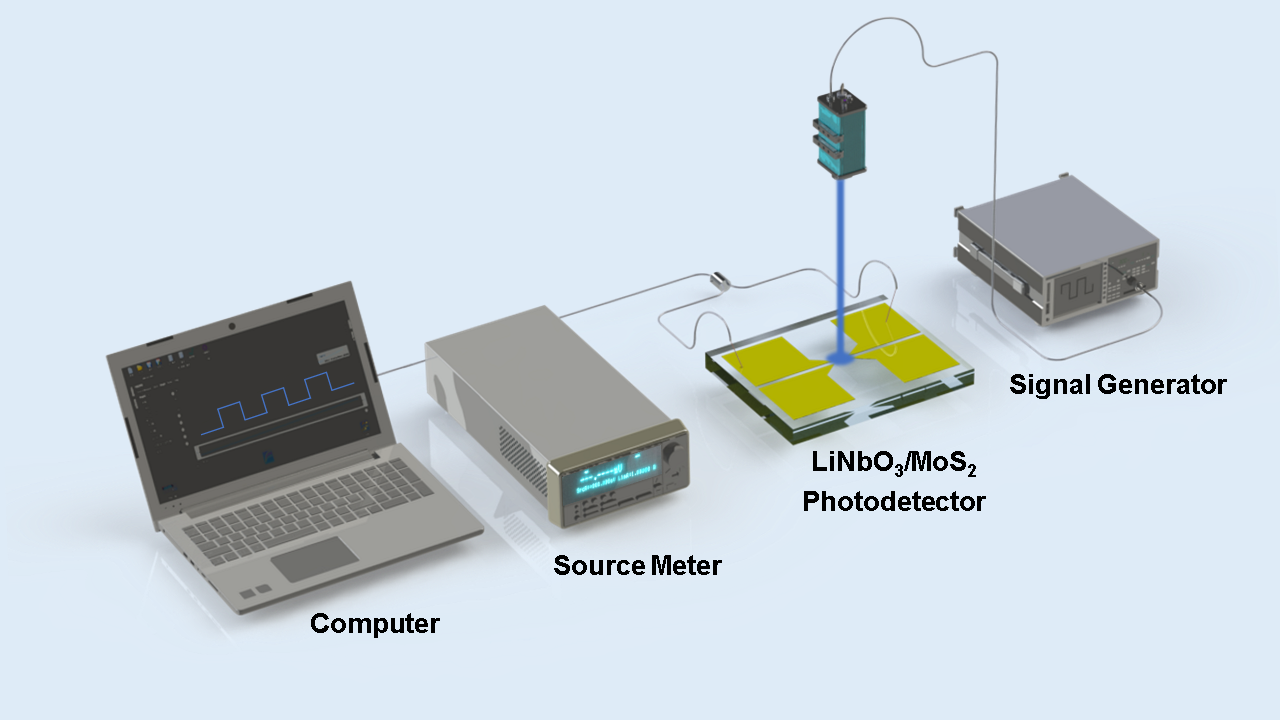


**Figure S15:** The system diagram for testing the performance of the MoS_2_/LiNbO_3_ photodetector mainly comprises a laser, source meter, signal generator, and computer.

| Wavelength (nm) | α | COD |
| --- | --- | --- |
| 365 | 0.39655 ± 0.00249 | 0.99929 |
| 405 | 0.4162 ± 0.00576 | 0.99656 |
| 462 | 0.36803 ± 0.01308 | 0.97777 |
| 532 | 0.41826 ± 0.00413 | 0.99825 |
| 660 | 0.37369 ± 0.00973 | 0.98795 |
| 808 | 0.44855 ± 0.0037 | 0.99878 |
| 1064 | 0.37657 ± 0.01084 | 0.99259 |

**Table S1:** The α value and COD of the fitted curve (*I*_ph_ ∝ *P*_in_^α^) of the extracted photocurrent.

**Reference**

1. Xu H, Wu J, Feng Q, Mao N, Wang C, Zhang J. High responsivity and gate tunable graphene-MoS_2_ hybrid phototransistor. Small. 2014;10(11):2300-6.
2. Choi MS, Qu D, Lee D, Liu X, Watanabe K, Taniguchi T, et al. Lateral MoS_2_ p-n Junction Formed by Chemical Doping for Use in High-Performance Optoelectronics. ACS Nano. 2014;8(9):9332-40.
3. Fan T, Xie Z, Huang W, Li Z, Zhang H. Two-dimensional non-layered selenium nanoflakes: facile fabrications and applications for self-powered photo-detector. Nanotechnology. 2019;30(11):114002.
4. Shen T, Li F, Zhang Z, Xu L, Qi J. High-Performance Broadband Photodetector Based on Monolayer MoS_2_ Hybridized with Environment-Friendly CuInSe_2_ Quantum Dots. ACS Appl Mater Interfaces. 2020;12(49):54927-35.
5. Yin Z, Li H, Li H, Jiang L, Shi Y, Sun Y, et al. Single-Layer MoS_2_ Phototransistors. ACS Nano. 2012;6(1):74-80.
6. Zhang X, Liao Q, Kang Z, Liu B, Ou Y, Du J, et al. Self-Healing Originated van der Waals Homojunctions with Strong Interlayer Coupling for High-Performance Photodiodes. ACS Nano. 2019;13(3):3280-91.
7. Sun M, Fang Q, Xie D, Sun Y, Xu J, Teng C, et al. Novel Transfer Behaviors in 2D MoS_2_/WSe_2_ Heterotransistor and Its Applications in Visible-Near Infrared Photodetection. Adv Electron Mater. 2017;3(4).
8. Zheng Z, Zhang T, Yao J, Zhang Y, Xu J, Yang G. Flexible, transparent and ultra-broadband photodetector based on large-area WSe_2_ film for wearable devices. Nanotechnology. 2016;27(22):225501.
9. Mitta SB, Ali F, Yang Z, Moon I, Ahmed F, Yoo TJ, et al. Gate-Modulated Ultrasensitive Visible and Near-Infrared Photodetection of Oxygen Plasma-Treated WSe_2_ Lateral pn-Homojunctions. ACS Appl Mater Interfaces. 2020;12(20):23261-71.
10. Chen X, Qiu Y, Yang H, Liu G, Zheng W, Feng W, et al. In-Plane Mosaic Potential Growth of Large-Area 2D Layered Semiconductors MoS_2_-MoSe_2_ Lateral Heterostructures and Photodetector Application. ACS Appl Mater Interfaces. 2017;9(2):1684-91.
